# Single-cell atlas of rhesus monkey testis reveals aging- and season-dependent regulation of spermatogenesis and microenvironment homeostasis

**DOI:** 10.7150/ijbs.134245

**Published:** 2026-07-20

**Authors:** Shengnan Wang, Yichao Wang, Li Wu, Xiang Shi, Yaping Yan, Lei Zhang, Weizhi Ji, Naixue Yang, Wei Si

**Affiliations:** 1 School of Basic Medical Sciences, Health Science Center, Dali University 671000, China.; 2 State Key Laboratory of Primate Biomedical Research, Institute of Primate Translational Medicine, Kunming University of Science and Technology, Kunming, Yunnan 650500, China.; 3 Yunnan Key Laboratory of Primate Biomedical Research, Kunming, Yunnan 650500, China.

## Abstract

Spermatogenesis is a highly orchestrated process that depends on precise coordination between germ cells and the testicular microenvironment. However, how intrinsic aging and seasonal transitions regulate this process in nonhuman primates remains poorly defined. Here we generate a single-cell transcriptomic atlas of the rhesus monkey testis spanning prepubertal, adult and aged stages across breeding and non-breeding seasons. We uncover pronounced seasonal oscillations in meiotic germ cells that coincide with remodeling of Sertoli-cell junctional programs, including tight- and adherens-junction components, supporting blood-testis barrier plasticity as a seasonally tuned layer associated with meiotic progression. Within the spermatogonial compartment, we identify a transcriptionally distinct, high-stemness undifferentiated spermatogonial state (U3) that is selectively enriched during the adult non-breeding season and progressively depleted with aging. U3 cells exhibit PDGF-associated signaling together with transcriptional programs linked to cytoskeletal organization and metabolic adaptation, consistent with a high-plasticity SSC state responsive to seasonal cues. Guided by these signatures, we establish a three-dimensional collagen-PDGF culture platform that enhances colony formation and supports extended propagation of rhesus SSCs in vitro. Finally, aging-associated shifts in testicular immune cell composition in rhesus monkeys were observed, including expanded CD3⁺CD4⁻CD8⁻ T-cell populations and pro-inflammatory macrophage profiles with diminished antigen-presentation signatures, and this phenotypic observation aligns with heightened inflammatory pressure on spermatogenic homeostasis. Collectively, this study delineates the age- and season-dependent regulation of spermatogenesis in rhesus monkeys, and provides new mechanistic insights into testicular aging and homeostasis.

## Introduction

Spermatogenesis is a highly orchestrated process that relies on continuous interactions between germ cells and somatic components within the testicular microenvironment^1^. In humans and non-human primates, testicular aging is characterized by progressive declines in spermatogenic output, Leydig and Sertoli cell dysfunction, and remodeling of the testicular microenvironment, underscoring the need to define aging-related cellular and molecular changes in primate testes at single-cell resolution. Age-related male reproductive decline is characterized by stem cell depletion, impaired somatic cell function, and disrupted immune homeostasis. Recent single-cell transcriptomics studies have delineated the cell composition of primate testes and revealed aging-associated alterations in both humans and macaques^2^-^4^. As a widely used experimental model, the rhesus monkey (*Macaca mulatta*) not only shares high physiological and genetic similarity with humans but has also evolved a distinct reproductive seasonality, characterized by pronounced fluctuations in spermatogenic activity, steroidogenesis, and immune composition^5^-^7^. These unique features, which remain underexplored at single-cell resolution, particularly in the context of aging, offer a valuable framework to elucidate how intrinsic aging and seasonal cues converge at the molecular and cellular levels to regulate testicular physiology^8^, ^9^. However, existing datasets on rhesus monkeys are predominantly cross-sectional, limiting the resolution needed to disentangle the complex interplay between intrinsic aging and extrinsic environmental factors, particularly reproductive seasonality. Consequently, the molecular basis by which intrinsic aging and reproductive seasonality jointly shape spermatogenesis, niche plasticity, and immune privilege remains largely undefined.

In the rhesus monkey, seasonal reproduction involves cyclical transitions between an active breeding phase, marked by elevated androgen production and robust spermatogenesis, and a quiescent non-breeding phase characterized by seminiferous epithelium regression, spermatogenic arrest, and altered immune activity. Within this dynamic testicular microenvironment, Sertoli cells provide structural and metabolic support essential for germ cell development^10^. A key feature of Sertoli cell function is the formation and dynamic remodeling of the blood-testis barrier (BTB)^11^, ^12^, which partitions the seminiferous epithelium into basal and luminal compartments via tight and adherens junctions, thereby maintaining testicular homeostasis and facilitating germ cell differentiation during meiosis^12^-^14^. Leydig cells, situated in the interstitial space between seminiferous tubules, serve as the primary source of testosterone^15^. Although testosterone may have limited effects on spermatogonial proliferation, it promotes meiotic progression^16^. Spermatogenesis is a highly intricate process that requires coordinated support from somatic cells both within and around the seminiferous tubules to establish an optimal microenvironment. Proper initiation of meiosis by spermatogonial stem cells (SSCs) along the basement membrane is critical for generating haploid spermatids. In addition to their differentiation potential, SSCs retain self-renewal capacity, underscoring the importance of SSC heterogeneity and niche remodeling in maintaining spermatogenesis under fluctuating conditions^17^. Despite extensive studies on BTB dynamics and SSC biology, the molecular mechanisms regulating BTB dynamics, somatic cell function, and SSC fate decisions, particularly under the influences of aging and seasonal transitions remain largely unresolved. The integrity of spermatogenesis is not only related to the testicular microenvironment but also directly intertwined with immune regulatory mechanisms^18^, ^19^. Consequently, its remodeling during aging or across breeding and nonbreeding seasons must be intrinsically linked to shifts in testicular immune modulation. Under physiological conditions, this immune privilege is sustained through a specialized microenvironment composed of activated (M2) macrophages, regulatory T cells, and tolerogenic dendritic cells, which collectively suppress inflammation and prevent autoimmune responses against germ cell antigens^20^. Aging shifts this balance toward a pro-inflammatory milieu characterized by M1 macrophage accumulation, increased antigen presentation, and NF-κB pathway activation, thereby promoting germ cell apoptosis and compromising niche integrity. Chronic low-grade inflammation further compromises SSC maintenance and accelerates degenerative changes in the seminiferous epithelium. However, the spatiotemporal dynamics, lineage plasticity, and intercellular communication of immune cells in the aging primate testis remain poorly characterized, particularly in the context of seasonal reproductive transitions.

How intrinsic aging and extrinsic seasonal cues modulate somatic cell function, influence BTB dynamics, shape SSC heterogeneity, and ultimately reprogram immune privilege remains unclear. To address these knowledge gaps, we applied single-cell transcriptomics to profile testes of rhesus monkeys across prepuberty, adult, and aged stages during breeding and nonbreeding seasons. We found that Sertoli cells exhibit season-associated remodeling of tight-junction programs that coincides with changes in meiotic germ-cell states, while SSCs balance meiosis initiation with stemness maintenance across distinct functional subpopulations. We identify a transcriptionally distinct, high-stemness undifferentiated spermatogonial state (U3) that is specifically enriched during the adult non-reproductive season and is characterized by activation of PDGF-associated pathway. Guided by this signature, we established a 3D collagen-PDGF composite scaffold system that enhances clonogenic formation and supports extended propagation of rhesus SSCs in* vitro*. In parallel, we observe aging-associated immune compositional changes in the testicular niche, including an increased abundance of a CD3⁺CD4⁻CD8⁻ T-cell population and pro-inflammatory macrophage programs. Notably, the pro-inflammatory macrophages (Mφ_SPAG16, M1-like) expand during the non-breeding season, consistent with heightened inflammatory pressure and reduced niche support. Together, this study provides a single-cell framework to interpret how intrinsic aging and seasonal transitions reshape spermatogonial, somatic and immune states in the testis of rhesus monkeys.

## Methods

### Animals and ethics

Six male rhesus monkeys (1-22 years) were included: prepubertal (1 year, n = 2), adult (6 years, n = 2), and aged (22 years, n = 2) groups. Testicular samples were collected following established protocols^21^. For seasonal comparison, each monkey contributed paired samples: one testis was collected during the non-breeding season (August) and the contralateral testis during the breeding season (December). To address potential effects of unilateral orchiectomy, we followed prior evidence showing that circulating gonadotropin and androgen levels exhibit only transient changes after hemicastration, with LH and testosterone returning to baseline within 48 h and no sustained alteration in FSH^22^. This paired design yielded two biological replicates (n = 2) for each age-season condition. All procedures were approved by the Institutional Animal Care and Use Committee of Kunming University of Science and Technology (authorization code: LPBR202104015) and were conducted in accordance with the *Guide for the Care and Use of Laboratory Animals* (8th edition).

### Isolation and preparation of single-cell suspensions from rhesus monkey testicular tissue

Testes were rinsed with PBS and minced into small pieces. The tissue fragments were digested with 5% type IV collagenase (Gibco, Life Technologies) at 37°C for 20 minutes. After two washes with PBS, digested samples were supplemented with 10% fetal bovine serum (FBS) to quench enzymatic activity and centrifuged at 400 g for 5 min. Cell pellets were resuspended in PBS containing 1% bovine serum albumin (BSA) and filtered through a 100 μm nylon cell strainer (B&D) to obtain single-cell suspensions.

### qRT-PCR analysis

Total RNA was extracted from testis tissue using TRIzol (Invitrogen, US) according to the manufacturer's instructions. Approximately 1µg of RNA was reverse-transcribed into cDNA using a commercial reverse transcription kit (TIANGEN, China). qPCR was performed using SYBR Green PCR master mix (Roche, Switzerland). Relative mRNA expression was normalized to β-actin and calculated using the 2-ΔΔCt method. PCR primer sequences are provided in Table S1.

### Immunofluorescence staining

Immunofluorescence staining was performed as described previously 7. Briefly, OCT-embedded sections (10 μm) were rinsed in distilled water, permeabilized with 0.4% Triton X-100 for 30 min, and washed three times in PBS. Sections were blocked with 10% donkey serum in PBS for 1 h at room temperature, incubated with primary antibodies overnight at 4 °C, and then incubated with fluorescence- conjugated secondary antibodies for 1 h at room temperature. Nuclei were counterstained with Hoechst 33342 (Thermo Fisher, H3570, 1:1000). Images were acquired on a Leica TCS SP8 DIVE confocal microscope. The primary antibodies used were: anti-SYCP3 (Novus, NB300-229, 1:100), anti-DDX4 (Abcam, Ab27591, 1:500), anti-Collagen IV (Millipore, AB769, 1:200), anti-p16 (SANTA CRUZ, sc56330, 1:100), and anti-SSEA4 (Millipore, 3849119, 1:100). The secondary antibodies were: rabbit anti mouse IgG (Abcam, ab150126, 1:500) and goat anti-rabbit (Abcam, ab150077, ab150080, and ab150079; 1:800).

### Electron microscopy

Testis tissue was excised into small pieces and fixed in 2.5% glutaraldehyde for 4 h at room temperature, followed by further fixation for 24 h at 4°C. Samples were rinsed three times (5 minutes each) in 0.1 M PBS and post-fixed in a 1:1 mixture of 2% osmium tetroxide and 3% potassium ferrocyanide in the dark for 2 h at 4°C. After three rinses in distilled water, samples were dehydrated through graded ethanol (30%, 50%, 70%, 95%, and 100%; two changes each, 10 min per change) and transitioned with 100% acetone (two changes, 10 min each). Samples were infiltrated with acetone: Epon 812 mixtures (3:1, 1:1, 1:3; 1 h each), followed by pure Epon 812 for 3 h, embedded, and polymerized at 65°C for 48 h. Ultrathin sections (70 nm) were cut with a 45° diamond knife, collected onto 100-mesh grids, and stained with 2% uranyl acetate in the dark for 8 min, followed by 2% lead citrate staining in the dark for 5 min. Sections were imaged on a Hitachi HT7800 transmission electron microscope.

### Flow cytometry

Single-cell suspensions (0.5×10^6^ cells) were incubated with fluorescence-conjugated antibodies for 30 minutes at 4 ℃. Prior to acquisition, dead cells and debris were removed using magnetic-bead-based cleanup. Unstained cells were used as a negative control, and single-stained CD3-PerCP-Cy5-5-A samples (BD, 555332) were prepared for compensation. For intracellular staining, cells were fixed and permeabilized using the BD Fixation/Permeabilization Solution Kit (BD, 554714) according to the manufacturer's instructions. Data were acquired on a flow cytometer (BD FACS Aria III) and analyzed with Flowjo VX software (10.8.1).

### SSCs culture

The culture medium for SSCs was composed of StemPro-34 SFM (Invitrogen, 10639-011), 10% fetal calf serum (FBS, HyClone), 1 mM penicillin (Lifetech Gibco), 2 mM L-Glutamine (Gibco, Cat# 11140-050), 0.1 mM β-mercaptoethanol (Sigma, M3148-25ML), 1 mM sodium pyruvate (Gibco, 11360070), 1 mM NEAA (nonessential amino acid, Gibco, 35050-061), 60 µM putrescine(Sigma, 51799-100MG), 40 ng/mL human GDNF (R&D, 212-GD), 50 ng/mL human PDGF (Stem cell, 78154), 10 µg/mL human biotin (Selleck, 58-85-5), 40 ng/mL VC (Vitamin C, Sigma, PHR1008-2G), 1 μM Melatonin (Stem cell, S2924), 10 ng/mL human EGF (Invitrogen, Cat# BMS320), 1000 U mouse LIF (Millipore, ESG1106), 2 ng/mL human bFGF (Millipore, GF003), and recombinant Wnt3a (R&D, 5036-WN-010). Wnt3a was prepared in 1% BSA. For 3D culture, 1×10^6^ cells were resuspended in 1 ml of RGF BME Type 2 (R&D, 3533-005-02) on ice. Aliquots (35 μL) were dispensed into wells to form spherical droplets allowed to gel at room temperature, and then overlaid with the SSCs culture medium. Culture was maintained at 37 °C in 5% CO_2_.

### Single-cell RNA-sequencing data processing

The raw data (fastq files) from single-cell RNA sequencing were pre-processed using the Cell Ranger Single Cell Software Suite (version 9.0.0) provided by 10× Genomics. The reads were aligned to the *Macaca mulatta* reference genome (Macaca_mulatta. ensembl. Mmul_10). Filtered gene-cell count matrices from each sample were imported into Seurat (v4.2.0) in R for downstream analysis. Quality control was first performed on each sample individually. Genes detected in fewer than three cells were excluded. Cells were removed if they contained fewer than 300 detected genes, more than 30,000 UMIs, or a mitochondrial transcript fraction greater than 3% (Table S2). Subsequently, we executed a global scaling normalization procedure using the “*NormalizeData*” function with the “*LogNormalize*” method for each sample. And the top 2,000 highly variable genes were identified using the “*FindVariableFeatures*” function. To integrate cells from different samples, we used the Reciprocal PCA (rpca) method of the “*FindIntegrationAnchors*” function to identify “anchors” between samples. We then integrated the datasets using the “*IntegrateData”* function with 30 dimensions, resulting in a Seurat object containing an integrated expression matrix for all cells. The integrated expression matrix was then scaled using “*ScaleData*” function, followed by principal component analysis (PCA) with *"RunPCA"*. Low-dimensional visualization was performed using Uniform Manifold Approximation and Projection (UMAP). Cell clusters were identified using Seurat's shared nearest-neighbor (SNN) graph-based clustering implemented in “*FindNeighbors*” and “*FindClusters*” functions.

To assess ambient RNA contamination, contamination scores were estimated for each sample using “decontX” (version 0.99.3). Clusters with a contamination rate greater than 0.9 were considered highly contaminated and were removed from subsequent analyses. Next, we performed preliminary cell annotation based on the expression of marker genes. We used the R package “DoubletFinder” (version 2.0.3) to estimate the doublet rate of the cells in the data based on the annotation results and sample information. Doublets identified by the algorithm were removed. After exclusion of contaminated clusters and predicted doublets, the dataset was reprocessed, resulting in a final high-quality dataset comprising 86,969 cells.

### Quantification of age/season enrichment across cell clusters

To quantify whether specific cell clusters were enriched or depleted under a given age or seasonal condition, we calculated the ratio of observed to expected cell numbers in each cell cluster across different tissues to get the ratio of observation to expectation (*Ro/e*). For a given cell cluster, *Ro/e* > 1 indicates relative enrichment, whereas *Ro/e* < 1 indicates relative depletion under that condition.

### RNA velocity, PAGA, and pseudotime analysis

RNA velocity analysis was performed using the scVelo Python package (version 0.3.2). Specifically, spliced and unspliced transcript count matrices were processed by filtering out genes with fewer than 20 shared counts, followed by normalization and the selection of the top 2,000 highly variable genes using *scv.pp.filter_and_normalize*. First- and second-order moments of cell neighborhood were computed across 30 nearest neighbors in the 30-dimensional principal component (PCA) space (*scv.pp.moments*). Gene-specific RNA velocities were then estimated using the stochastic model (*scv.tl.velocity*), which captures steady-state assumptions while accounting for intrinsic expression variability. A velocity graph was constructed based on cosine correlations of cell-to-cell transitions (*scv.tl.velocity_graph*), and the resulting velocity vectors were projected onto UMAP embeddings to visualize cellular dynamics as streamlines (*scv.pl.velocity_embedding_stream*). Furthermore, to accurately capture fine-grained developmental trajectories across different physiological conditions, PCA and nearest-neighbor graphs (n_pcs = 30, n_neighbors = 30) were independently re-calculated for each subgroup prior to subgroup-specific velocity estimation. Finally, the temporal progression of cells was inferred using scVelo's pseudotime computation (*scv.tl.velocity_pseudotime*). To examine coarse-grained relationships among spermatogonial subpopulations, partition-based graph abstraction (PAGA) analysis was performed using Scanpy (version 1.10.1). Specifically, neighborhood graphs were reconstructed from diffusion map coordinates with n_neighbors set to 10. Cluster-level connectivity was then estimated using the default PAGA model (v1.2) based on these graphs. This approach was used to summarize the connectivity structure among undifferentiated, differentiating, and differentiated spermatogonial states and to support trajectory interpretation at the cluster level.

### Transcription factor regulon activity

Transcription factor regulon activity was assessed using “pySCENIC” (version 1.0.1). Regulons were initially identified by examining the coexpression patterns between transcription factors and their target genes. This was followed by a motif analysis to confirm the regulatory interactions. The activity of each regulon in individual cells was quantified using the AUCell algorithm, which computes a score between 0 and 1, indicating the regulon's activity level. The “ComplexHeatmap” (version 2.18.0) package was utilized to generate heatmaps of the AUC values for all regulators with row clustering method performed using the “complete” linkage method to form five distinct clusters. Subsequently, the target genes within each cluster were subjected to enrichment analysis using the Gene Ontology (GO) and Kyoto Encyclopedia of Genes and Genomes (KEGG) databases via the msigdbr package. Pathways that were significantly enriched and exhibited unique functions were selected for presentation.

### Cell-type prioritization analysis with Augur

Cell types of germ cells were ranked using the R package “Augur” (version 1.0.3), which is an analytical framework that prioritizes cell types most responsive to biological perturbations or conditions in single-cell genomics data. Briefly, the method selects the most variable genes from a set of single-cell data, withholds a subset of cells for testing, trains classifiers with the remaining cells for each cell type, and predicts the withheld labels using the trained model. This process is repeated over several iterations, and an AUC is reported for each iteration and cell type. An AUC of 1 indicates a perfectly predictive model, while an AUC of 0.5 indicates a model that predicts no better than random guessing. For AUC analysis of germ cells across different ages or seasons, we used the default Augur parameters.

### Identification of differentially expressed genes (DEGs)

Differentially expressed genes (DEGs) were identified using the Wilcoxon rank-sum test via the “*FindMarkers*” function in Seurat. Genes detected in at least 10% of cells in either comparison group were included in the analysis (min.pct = 0.1). P-values were adjusted for multiple testing using the Bonferroni correction, and genes with an adjusted P value < 0.05 were considered significantly differentially expressed genes. To assess the biological functions of differentially expressed genes in each comparison, we performed enrichment analysis of gene ontology (GO) biological process (BP) using “clusterProfiler” R package (version 4.10.1).

### Cell-cell communication analysis

Intercellular communication networks were inferred using the R package CellChat (v2.1.2). For each analysis group, a CellChat object was constructed from normalized gene expression data. We utilized the human ligand-receptor interaction database (CellChatDB.human), focusing specifically on "Secreted Signaling" pathways. To account for potential signaling dropout, expression data were projected onto a protein-protein interaction network (PPI.human) before computing communication probabilities. To ensure statistical reliability, any interaction involving cell types with fewer than 10 cells was filtered out. Signaling pathway-level communications were subsequently calculated using the *computeCommunProbPathway* function. Finally, the global communication network was aggregated via *aggregateNet* to quantify total interaction strengths and the number of connections between cell types.

### Empirical cumulative distribution function

The empirical cumulative distribution function (ECDF) represents the cumulative distribution of each value in a given dataset. It was visualized by plotting the proportion of observations in the dataset that were less than or equal to a given value against the total number of observations. Using the ggecdf function from the ggpubr R package (version:0.6.0), we plotted the distribution curves of germ cells along pseudotime under each age-season condition to analyze the distribution characteristics and compare their differences.

### Correlation analysis of SSC subpopulations across species

We obtained the human dataset from the Gene Expression Omnibus (GEO) database under accession number GSE142585 and the mouse dataset under accession number GSE112393. Both datasets were downloaded directly from the GEO Data Portal (https://www.ncbi.nlm.nih.gov/geo/) and used for subsequent analyses. Human and mouse genes were first converted to macaque orthologs using the homologene function. Pearson correlation coefficients were then calculated using the cor function between SC subpopulations from monkey and human datasets, as well as between monkey and mouse datasets. Higher Pearson correlation coefficients indicate stronger transcriptional similarity between corresponding SC subpopulations.

### Statistical analyses

Statistical analyses were performed using the R “ggpubr” package (version 0.6.0). Two-group comparisons were performed using two-sided Student's t-tests unless otherwise noted. Details of statistical tests, adjusted P-values, and sample definitions are provided in the relevant figure legends and method descriptions.

## Results

### Testicular tissue in rhesus monkeys undergoes seasonal shifts and age-related degeneration

**Figure 1:**
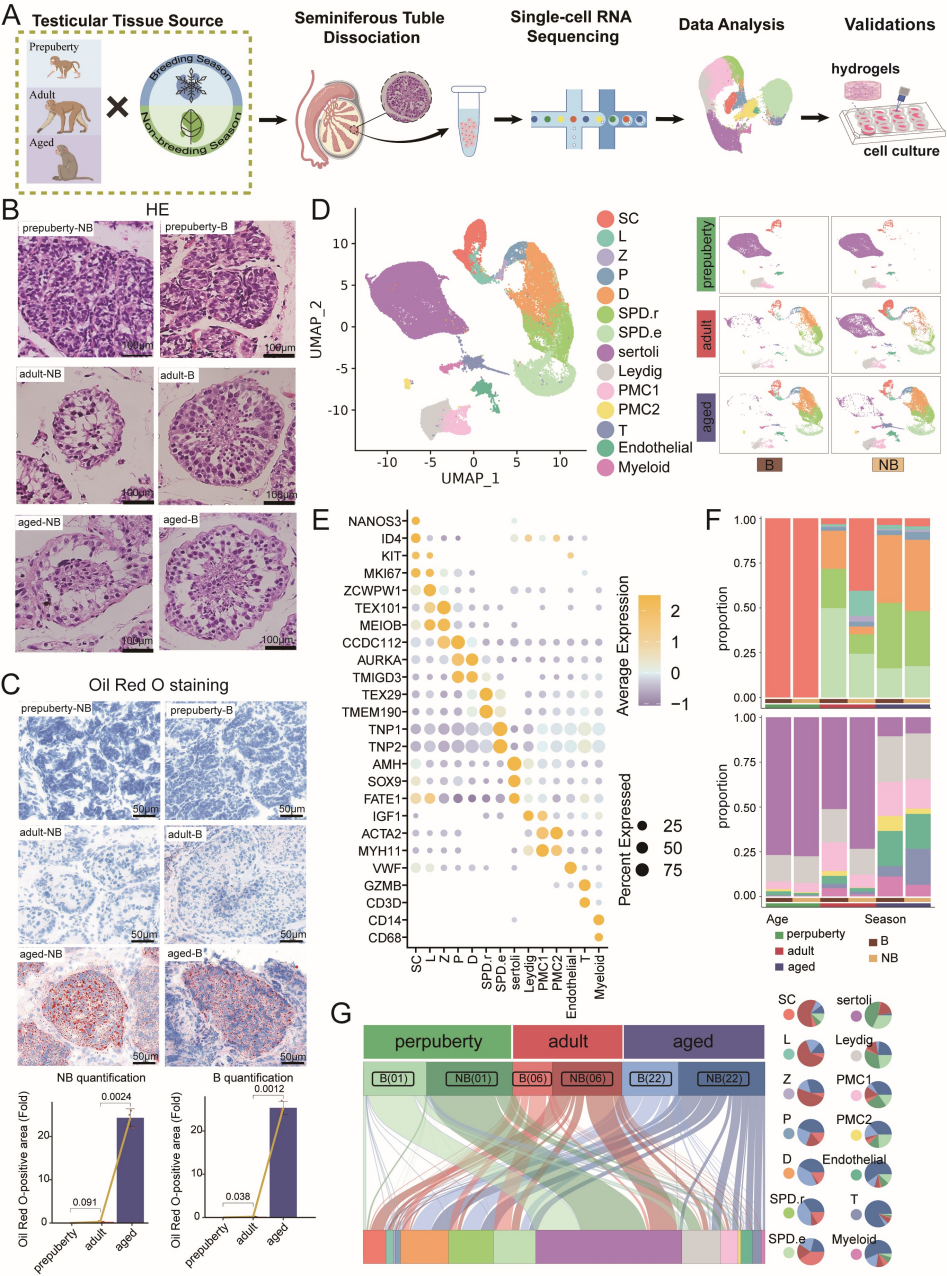
** Single-cell transcriptomic profiling revealed seasonal shifts and age- associated changes in rhesus monkey testes. A** Flowchart of the experimental design depicting testicular aging and reproductive seasons in rhesus monkeys. **B** Representative H&E staining of testicular sections across ages and seasons. Scale bars, 100 μm. **C** Oil Red O staining of testicular sections from prepuberty, adult and aged monkeys during the non-breeding (NB) and breeding (B) seasons. Upper panel: representative images; Lower panel: quantification of Oil Red O-positive area in NB and B samples, respectively, presented as fold changes. Data are shown as means ± SEM. Scale bars, 50 μm. **D** UMAP plot showing testicular cell types. Left panel: annotation of 14 distinct cell types in monkey testes. Right panel: distribution of different cell types across age and seasonal groups. **E** Dot plot showing cell type canonical marker gene expression. Dot size represents the proportion of cells expressing each gene; Color indicates average expression level. **F** Bar plots showing the proportions of germ cells (top) and somatic cells (bottom) across age and seasonal conditions. **G** The Sankey diagram (left) showing the flow from age and seasonal categories to specific cell types. Pie charts (right) summarize the proportional distribution of age and seasonal groups within each cell type.

To dissect seasonal and age-associated dynamics of testicular architecture, bilateral testicular samples were collected from six rhesus macaques during the breeding and non-breeding seasons, spanning prepubertal (1 year, n = 2), adult (6 years, n = 2), and aged (22 years, n = 2) stages (Fig. 1A). Hematoxylin and eosin (H&E) staining showed that prepubertal testes exhibited immature cord-like structures lacking an apparent laminae or lumens, with no discernible seasonal variation. This morphology closely resembles testicular structures observed in fetal or early postnatal mice and juvenile humans^3^. In adults, seminiferous tubules were enlarged and densely populated with spermatogenic cells during the breeding season, but became reduced in diameter with markedly fewer germ cells during the non-breeding season (Fig. 1B). A similar seasonal pattern persisted in aged testes; however, spermatogenic cells content was markedly decreased even in the breeding season (Fig. 1B).

Consistent with age-associated degeneration, immunofluorescence revealed increased *P16*-positive cells in aged testes^23^, ^24^ (Fig. S1), and Oil Red O staining showed elevated lipid deposition, a classical histopathological feature of testicular aging^25^ (Fig. 1C). In addition, collagen IV staining indicated thickening of the seminiferous-tubule basement membrane in aged samples (Fig. S1). This extracellular matrix remodeling likely increases local tissue stiffness, potentially impairing nutrition and metabolite exchange within the seminiferous tubules^2^. Together, these histological results demonstrate pronounced seasonal remodeling of seminiferous tubules in adults and progressive degenerative changes with aging in rhesus monkey testes.

### Single-cell atlas uncovers seasonal and age-dependent changes in testicular cell composition

We performed single-cell RNA sequencing (scRNA-seq) on testicular samples from six rhesus monkeys across three age groups, including prepubertal (1 year, n = 2), adult (6 years, n = 2), and aged monkeys (22 years, n = 2), with paired collections in the breeding and non-breeding seasons. A total of 86,969 single-cell transcriptomes were obtained after quality control. Using Uniform Manifold Approximation and Projection (UMAP) and unsupervised clustering analysis, 14 major cell types based on canonical lineage markers were identified, including: spermatogonial cells (SC: ID4, NANOS3, KIT, MKI67), leptotene spermatocytes (L: ZCWPW1), zygotene spermatocytes (Z: TEX101, MEIOB), pachytene spermatocytes (P: CDC112), diplotene spermatocytes (D: AURKA, TMIGD3), round spermatids (SPD.r: TEX29, TMEM190), elongated spermatids (SPD.e: TNP1, TNP2), Sertoli cells (Sertoli: AMH, SOX9, FATE1), Leydig cells (IGF1), peritubular myoid cells (PMC: ACTA2, MYH11), endothelial cells (VWF), T cells (CD3D), and myeloid cells (CD14, CD68) (Fig. 1D, E)^3^, ^17^, ^26^, ^27^. Comparative analysis revealed dynamic changes in the proportions of cell types of testes across age and seasons (Fig. 1F, G). Notably, spermatogonial populations declined with age, whereas immune cell populations particularly T cells and myeloid cells expanded significantly in aged testes, indicating a shift toward a pro-inflammatory testicular microenvironment. In adults, meiotic germ cells exhibited synchronized seasonal shifts, while Sertoli cells showed distinct sensitivities to both aging and seasonal cues. Collectively, these results establish a single-cell atlas of rhesus monkey testes, demonstrating cellular remodeling associated with aging and reproductive seasonality.

### Transcriptomic profiling reveals seasonal- and age-dependent transcriptional dynamics in rhesus monkey testes

**Figure 2:**
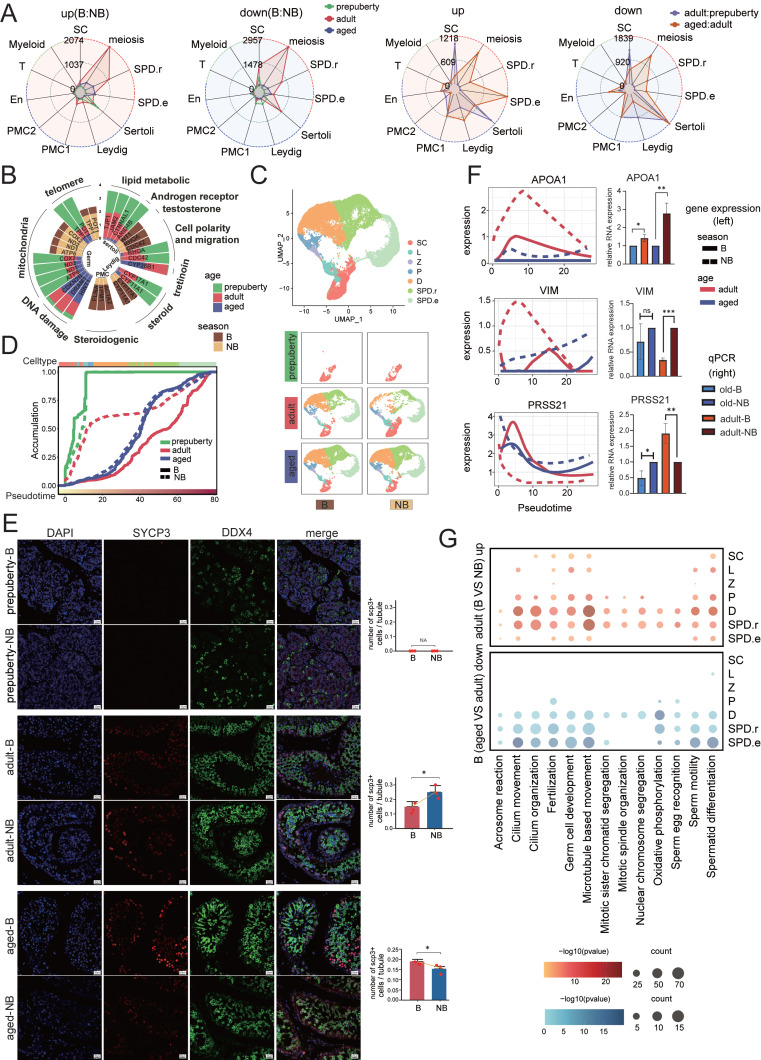
** Adult testicular meiosis in rhesus monkeys exhibits seasonal transcriptional oscillations aligned with the reproductive cycle. A** Radar plot displaying the numbers of DEGs across cell types under different comparisons. Left panel: upregulated (left) and downregulated (right) DEGs in breeding season versus non-breeding season across age stages. Right panel: upregulated (left) and downregulated (right) DEGs during development and aging. The numerical labels indicate the scale of DEG counts. **B** Circular bar plots showing scaled expression of representative functional gene categories across cell types under indicated age or seasonal conditions. The length of each bar represents the scaled expression level. Arcs indicate functional gene categories, while inner ring indicates cell types. **C** UMAP plot illustrating the cell subtypes of spermatogenic cells. Upper panel: cell type annotation; lower panel: distribution across age groups and reproductive seasons. **D** Empirical Cumulative Distribution Function (ECDF) plot displaying the distribution of spermatogenic cells along pseudotime progression. Curves represent the cumulative proportion of cells reaching each pseudotime point under different age and seasonal conditions. **E** Immunofluorescence staining of seminiferous tubules showing DAPI (blue), DDX4 (green) and SYCP3 (red). Representative images are shown in the upper panels, and quantification (means ± SEM) is shown in the lower panels. Positive SYCP3 positive area is quantified as fold changes. Scale bars, 20 μm.** F** Line plot modules displaying the smoothed expression of key regulatory genes during meiosis along pseudotime (left panel), and their corresponding qPCR validation results (right panel). **G** GO terms enrichment analysis of DEGs in germ cell types. Orange indicates pathways upregulated in breeding versus non-breeding seasons in adults, whereas blue indicates pathways downregulated in aged versus adult. Dot size represents the number of genes assigned to each GO term. Color intensity indicates statistical significance, with p-values calculated using the hypergeometric distribution.

To investigate how the testicular microenvironment responds to seasonal shifts and aging, we performed differential gene expression analysis across germ, somatic, and immune cell compartments. Transcriptomic comparisons revealed broad seasonal- and age-related transcriptional changes. Notably, spermatocytes undergoing meiosis (L/Z/P/D spermatocytes) exhibited the most pronounced seasonal variation in adults, a pattern absent in prepubertal and aged groups (Fig. 2A). During testicular aging, germ cells accumulated DNA damage accompanied by a progressive decline in the mitochondria-telomere axis across both breeding and nonbreeding seasons. Sertoli cells demonstrated marked downregulation of metabolic and endocrine pathways, including lipid metabolism, testosterone signaling, and retinoic acid pathways, while sex hormone levels and cell polarity/migration appeared to be coordinately regulated by dual chrono-regulatory factors. In parallel, Leydig cells underwent dynamic remodeling of steroidogenic pathways across age and seasons, indicating that environmental rhythms and intrinsic aging jointly disrupt testicular homeostasis (Fig. 2B). Subsequently, refined sub-clustering of germ cells followed by pseudotime analysis revealed a continuous developmental trajectory from SC to elongated spermatids (Fig. 2C). Seasonal pseudotime divergence was observed exclusively in adult germ cells, with the most evident shift during meiosis (Fig. 2D). Notably, meiotic cells aligned earlier along the differentiation trajectory in the non-breeding season, supporting a potential link to seasonal delay or partial arrest in meiotic progression (Fig. S2A). Moreover, immunofluorescence staining validated the spatiotemporal localization of this developmental transition node, further supporting seasonal regulation of meiotic progression (Fig. 2E).

We identified 467 genes exhibiting dynamic expression during meiosis, categorized into three modules with distinct functional signatures influenced by seasonal reproductive cues (Fig. S2B). Module 1 delineated pathways primarily engaged during non-breeding season, including endothelial cell migration and cell-matrix adhesion. Module 2 comprised genes regulated during the transition from non-breeding to breeding season, and was enriched in pathways such as protein kinase C signaling, propionate metabolism, calcium signaling pathways, and cilia/flagella-dependent cell motility. These pathways likely reflect dynamic chromatin remodeling associated with germ cell division, with propionate metabolism and PKC-calcium signaling coordinating key meiotic processes. Module 3 contained genes progressively upregulated along the differentiation trajectory, particularly in cells undergoing advanced spermatogenesis, and was enriched in pathways associated with germ cell development and sperm nuclear differentiation, indicating preprogrammed gene expression to support fertilization. In *vitro* validation further confirmed elevated expression of adherent ligation-related genes such as *APOA1* during meiosis in the no-breeding season. *PRSS21*, a marker of testicular integrity 28, and Vimentin (*Vim*), an essential desmosome-like connexin in the BTB, were upregulated during the non-breeding season and showed age-related variation, as confirmed by qPCR (Fig. 2F). Collectively, these results suggest that meiosis in adult testes during the non-breeding season proceeds within a context of tight BTB regulation, potentially reflecting an adaptive mechanism to preserve germ cell integrity during seasonal reproductive quiescence.

Functional enrichment analysis further demonstrated that adult germ cells during the breeding season upregulated multiple core biological pathways associated with energy metabolism-associated processes (oxidative phosphorylation), regulatory mechanisms of germ cell development (spermatogonial differentiation and germ cell maturation), core mitotic machinery (sister chromatid segregation, spindle organization, and nuclear chromosome partitioning), motility-related pathways (cilia-driven movement, microtubule-based locomotion, and sperm motility), and critical reproductive events (acrosome reaction, fertilization, and gamete recognition). Notably, these activities declined progressively with age (Fig. 2G), highlighting dysregulation of temporal coordination as a potential driver of reproductive degeneration. Together, the seasonal dynamics during meiosis may represent a key entry point for understanding the evolutionary strategies in primate reproductive adaptation.

### Seasonal coordination between meiotic dynamics and Sertoli cell tight junction remodeling in adult rhesus monkey testes

Testicular somatic cells, including Sertoli cells (nutritional support and blood-testis barrier formation), Leydig cells (testosterone production to support masculinization and spermatogenesis), and peritubular myoid cells (sperm transport and microenvironment regulation), collaboratively support spermatogenesis and maintain testicular homeostasis. Somatic cell populations were identified based on established lineage-specific markers, including *AMH* for prepubertal Sertoli cells (Sertoli.p), *SOX9*^17^ for adult Sertoli cells (Sertoli.a) (Fig. S3A-C), and *DLK* and *IGF*^29^ for leydig cells. Leydig cells were further subdivided into four distinct subtypes (Leydig1-4) characterized by the expression of* PRKG1*, *FSHR*, *PRM1*, and *SKAP1* (Fig. S3F-H). Peritubular myoid cells (*ACTA2*, *MYH11*) were subdivided into PMC1-1 (*PI4K2A*), PMC1-2 (*IGF2*), PMC2-1 (*DLC1*), PMC2-2 (*RERGL*), and PMC2-3/PMC2-4 (*TJP1*) clusters (Fig. S3K-M)^26^.

Both aging and seasonal shifts reshaped testicular microenvironment through functional remodeling of Sertoli cells (Fig. S3D, E), Leydig cells (Fig. S3I, J), and peritubular myoid cells (Fig. S3N, O). During the breeding season, they preferentially activated spermatogenesis-related pathways and suppressed stress-response pathways. Leydig cells exhibited subtype-specific functions: Leydig-1/2 supported developmental steroidogenesis, Leydig-3 maintained cellular homeostasis and exhibited seasonal responsiveness, and Leydig-4 coordinated endocrine-immune interactions. Among PMCs, PMC1-1 displayed an age-related decline in contractility, impairing sperm transport, while PMC1-2 dynamically regulated hemodynamics for seasonal adaptation. This spatial and functional compartmentalization between seminiferous epithelium and interstitium ensures the integrated regulation of efficient spermatogenesis across age and reproductive seasons.

**Figure 3:**
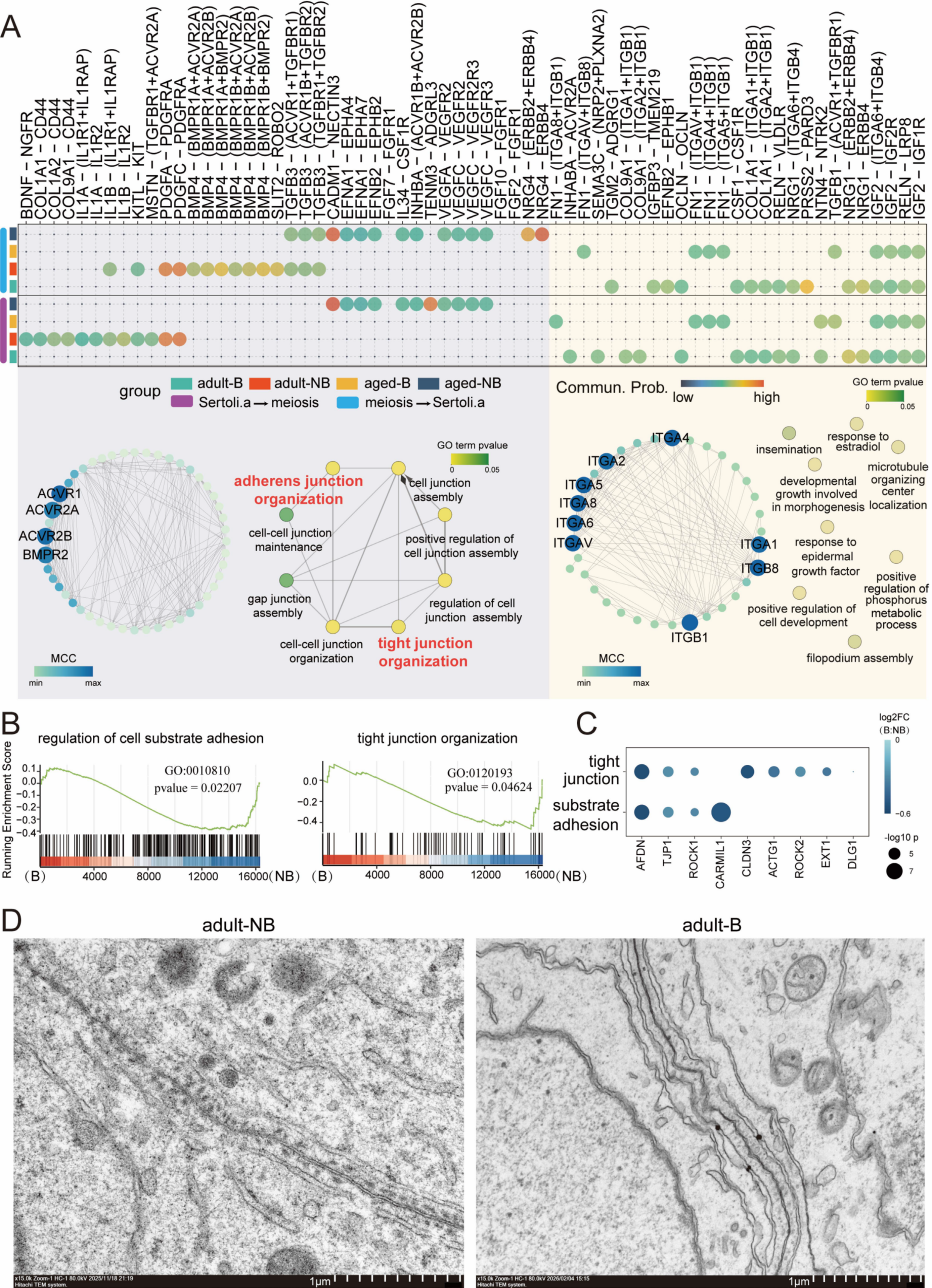
** Sertoli cells regulate seasonal meiotic dynamics through tight junction-mediated blood-testis barrier remodeling. A** Bubble heatmap showing ligand-receptor interaction pairs between Sertoli.a cells and meiosis germ cells across age and seasonal conditions. Dot color gradient reflects interaction strength. Network representations visualize enriched GO terms of significant interaction pairs in the non-breeding season (left, grey) and breeding season (right, light yellow). The accompanying protein-protein interaction (PPI) network visualizes the corresponding gene pairs identified in the bubble plot. Node color and size represent the Matthews correlation coefficient (MCC), reflecting the importance of each gene in the network. **B** GSEA showing enrichment of "regulation of cell substrate adhesion" in Sertoli. a cell (left) and "tight junction organization" in meiosis cells (right). Genes were ranked by log_2_(fold change) (breeding versus non-breeding seasons). **C** Dot plot displaying log_2_(fold change) (breeding versus non-breeding seasons) of representative genes associated with “tight junction organization” and “regulation of cell substrate adhesion” in Sertoli.a cells (top) and meiotic cells (bottom). Dot size indicates the number of genes contributing to each term; dot color indicates statistical significance. **D** Representative transmission electron microscopy images showing tight junction ultrastructure between adjacent Sertoli cells at the basal region of seminiferous tubule during the non-breeding and breeding seasons. Scale bars, 1 μm.

Intercellular crosstalk analysis further revealed that somatic-germ cell interactions peaked in adults but weakened with aging (Fig. S4A). Sertoli-meiotic cell interactions predominated during the non-breeding season and showed age-dependent decline in both ligand-receptor specificity and synchronized function (Fig. S4B-D). Given the central role of Sertoli-meiotic cell crosstalk in spermatogenesis, we performed targeted ligand-receptor analyses to elucidate the mechanisms underlying seasonal regulation and aging-related alterations. During the non-breeding season in adult testes, we identified interaction pairs associated with Sertoli-meiotic cell communication. Among them, COL1A1-CD44, a dominant adhesion complex, with CD44 as the principal adhesion receptor^30^, emerged as the predominant Sertoli-meiotic interactor. In contrast, during the breeding season, the NRG1-ERBB2/ERBB4 axis was enriched, consistent with the NRG1-androgen receptor axis driving spermatogonial stem cell renewal and meiotic progression^31^. Functional enrichment analysis further revealed that cell-cell interaction pairs were enriched in tight junction and cell-cell adhesion pathways during the non-breeding season, whereas breeding season related interactions were preferentially involved in steroid hormone biosynthetic, process cellular response to testosterone stimulus, and positive regulation of hormone metabolic process (Fig. 3A).

In our study, GSEA revealed significant upregulation of tight junction pathways and extracellular matrix adhesion regulators in both meiotic germ cells and Sertoli cells during the non-breeding season, suggesting that tight junction-mediated signaling is closely linked to seasonal meiotic cyclicity in adult monkeys (Fig. 3B, C). Electron microscopy further showed that TJ structures formed a continuous sealing network during the non-breeding season, consistent with reinforced BTB integrity. ‌In contrast‌, during the breeding season, TJ unstructured appeared looser and less organized, suggesting partial junctional disassembly and reduced sealing continuity (Fig. 3D). Concomitantly, seasonal changes in transmembrane junctional components may modulate paracellular permeability to support stage-specific exchange demands. Collectively, these observations indicate that seasonal BTB remodeling exhibits structural and molecular features correlated with adult meiotic dynamics.

### Seasonally enriched U3 SSC state is supported by PDGF signaling and enables prolonged rhesus SSC propagation in *vitro*

The BTB facilitates meiotic germ cell differentiation and dynamically modulates the SC microenvironment to maintain the stem cell pool. Augur analysis identified SC as one of the testicular populations most responsive to both aging and seasonal shifts (Fig. S5A). Consistently, DEGs analysis revealed that meiosis spermatocytes (L/Z/P/D) exhibited the strongest seasonal transcriptional variation, whereas SC were more prominently affected by aging while also displaying sensitivity to seasonal changes (Fig. 2A).

**Figure 4:**
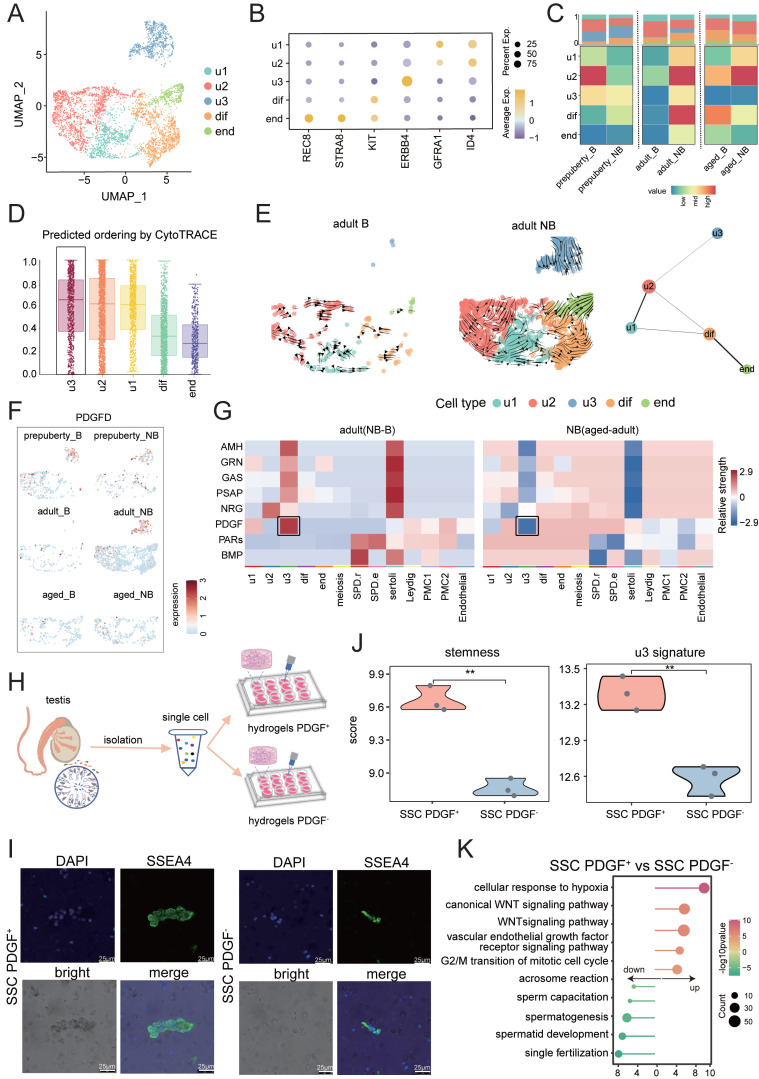
** Seasonally enriched U3 SSCs exhibit PDGF-associated programs. A** UMAP visualization of SSC subpopulations, showing undifferentiated SSC subclusters (U1-U3), differentiating spermatogonia (dif), and differentiated spermatogonia (end). **B** Dot plot showing the expression patterns of cell subtype-specific marker genes in SSCs. Dot size indicates the fraction of expressing cells, and color indicates scaled average expression. **C** Heatmap displaying Ro/e values for each SSC subtype across age and seasonal conditions, with the accompanying bar plot on top indicating subtype proportions. **D** Boxplot comparing CytoTRACE scores across SSC subpopulations, with higher scores indicating greater stem-like potential. **E** RNA velocity and PAGA analyses of relationships among U1-U3. The left and middle panels show RNA velocity-inferred dynamics of SSC states in adult breeding and non-breeding testes. The PAGA graph (right panel) illustrates connectivity among U1, U2, U3, differentiating spermatogonia (dif), and differentiated spermatogonia (end). Node size is proportional to cell number, and edge thickness of lines between nodes reflects connection strength.** F** UMAP plot displaying PDGFD expression, highlighting enrichment of PDGF-associated signaling in U3 cells during the adult non-breeding season. **G** Heatmap depicting pathway-level interaction strength between breeding and non-breeding seasons across various cell types in adult and aged testicular tissues. **H** Schematic flowchart depicting the 3D collagen-PDGF hydrogel culture system for rhesus SSCs. **I** Immunofluorescence staining of cultured rhesus SSCs colonies showing DAPI (blue) and SSEA4 (green). Bright-field image shown in gray. Scale bars, 25 μm. **J** Transcriptomic evaluation of cultured SSCs under indicated culture conditions. Left panel: stemness scores; right panel: expression of U3-specific signature genes. **K** Bar plots showing enriched GO terms enriched in PDGF-treated cultured SSCs compared with PDGF-free controls.

Unsupervised clustering resolved three major spermatogonial populations corresponding to successive developmental stages: undifferentiated SSCs (U1-U3: *ID4*^+^, *GFRA1*^+^, *ERBB4*^+^), differentiating spermatogonia (dif: *KIT*^+^), and differentiated spermatogonia (end: *STRA8*^+^, *REC8*^+^) (Fig. 4A, B and Fig. S5B). Within the undifferentiated spermatogonial compartment, U3 represents a transcriptionally distinct, high-stemness undifferentiated spermatogonial state with age- and season-related dynamics: it remained stable across seasons in prepubertal testes, was selectively enriched in adults during the non-breeding season, and became scarce in aged testes (Fig. 4C). Among all SSC subpopulations, U3 cells exhibit the highest CytoTRACE score (Fig. 4D), supporting a high-plasticity SSC state that is preferentially present during the adult non-breeding season and progressively depleted with aging. These findings underscore pronounced age- and season-dependent heterogeneity within the SSC compartment, with U3 representing a high-stemness undifferentiated state that is progressively lost with aging.

To further characterize relationships among U1-U3, we integrated RNA velocity and PAGA analyses. RNA velocity revealed prominent directional flux within the U1/U2 compartment, whereas U3 showed minimal connectivity to U1/U2 and appeared as a partially decoupled state, most evident in adult testes during the non-breeding season (Fig. 4E). These patterns support the interpretation that U3 represents a distinct SSCs state associated with the non-breeding niche context rather than a simple intermediate along the main U1/U2 differentiation stream. Subsequent PAGA analysis mapped interconnections among the undifferentiated SSC subpopulations and highlighted a linear progression toward differentiating and differentiated spermatogonia, with U3 occupying an upstream position, suggesting transcriptional heterogeneity within the undifferentiated pool and U3-linked entry point into the differentiation trajectory (Fig. 4E). Functional enrichment analysis further showed that U3 cells downregulated differentiation-associated pathways relative to U1/U2 (Fig. S5C). Pathway analysis of U3-specific genes and their downstream targets highlighted prominent enrichment in transforming growth factor beta (TGF-β), regulation of microtubule cytoskeleton organization, RAS protein signal transduction, and canonical Wnt signaling pathway (Fig. S5D). Together with the RNA velocity and PAGA results, these data are consistent with U3 representing a high-stemness undifferentiated spermatogonial state with low differentiation priming, rather than an actively cycling intermediate along the U1/U2 stream. Cross-species comparison further revealed low transcriptional similarity between rhesus monkey U3 cells and reported human^32^ or murine^33^ SSC counterparts (Fig. S5E, F, Table S3), supporting lineage specificity of this rhesus monkey SSC state. Notably, in adult testes during the non-breeding season, U3 cells showed coordinated upregulation of PDGFD (Fig. 4F), indicating an association between PDGF-related signaling and U3-like transcriptional programs. Guided by this signature, we developed a collagen-PDGF hydrogel culture platform to support rhesus SSC maintenance, which markedly enhanced clone formation and sustained proliferation >14 days (Fig.4G-I). Transcriptomic profiling of cultured SSCs further showed that PDGF supplementation increased stemness-associated activity scores and induced expression of U3-characteristic genes (Fig.4J). Consistently, pathway enrichment analysis demonstrated upregulation of vascular endothelial growth factor receptor (VEGFR) signaling and canonical Wnt signaling, accompanied by downregulation of spermatogenesis- associated pathways (Fig.4K). Together, these results identify a seasonally enriched U3 spermatogonial state with PDGF-associated transcriptional features. In *vitro* culture experiments further show that PDGF-containing 3D culture conditions support prolonged rhesus SSC propagation and partially recapitulate U3-like molecular features in vitro.

### Testicular immune deterioration: aging-associated shifts in testicular immune cell composition in rhesus monkeys

The testicular immune system maintains a distinctive balance between immune privilege and pathogen-responsive capacity. Immune cells support testicular homeostasis by dynamically balancing pro-inflammatory and immunoregulatory states, thereby shaping an immune-protected microenvironment. Across the lifespan, we observed a pronounced age-associated accumulation of T cells within the testis (Fig. 1D, E). Sub clustering and spatial phenotyping further revealed preferential enrichment of a CD3D⁺ CD4⁻CD8⁻ double-negative T cell population (DNT) in aged testes (Fig. S6A, B). Flow cytometry analysis confirmed age-dependent differences in DNT abundance across prepubertal, adult, and aged testes during both breeding and non-breeding seasons (Fig. S6C). Subsequent qPCR validation supported the specific expansion of this testis-resident DNT population with aging (Fig. S6D).

Functional enrichment analysis indicated coordinated downregulation of pathways associated with T cell activation, antigen receptor-mediated signaling, adaptive immune regulation, immune effector processes, and T cell chemotaxis in aged testes (Fig. S6E). Given prior evidence that DNT cells can suppress the proliferation of conventional T and B lymphocytes^34^, their accumulation in aged testes is consistent with a shift toward an immunoregulatory state that may contribute to compromised immune surveillance in the aging testicular niche.

**Figure 5:**
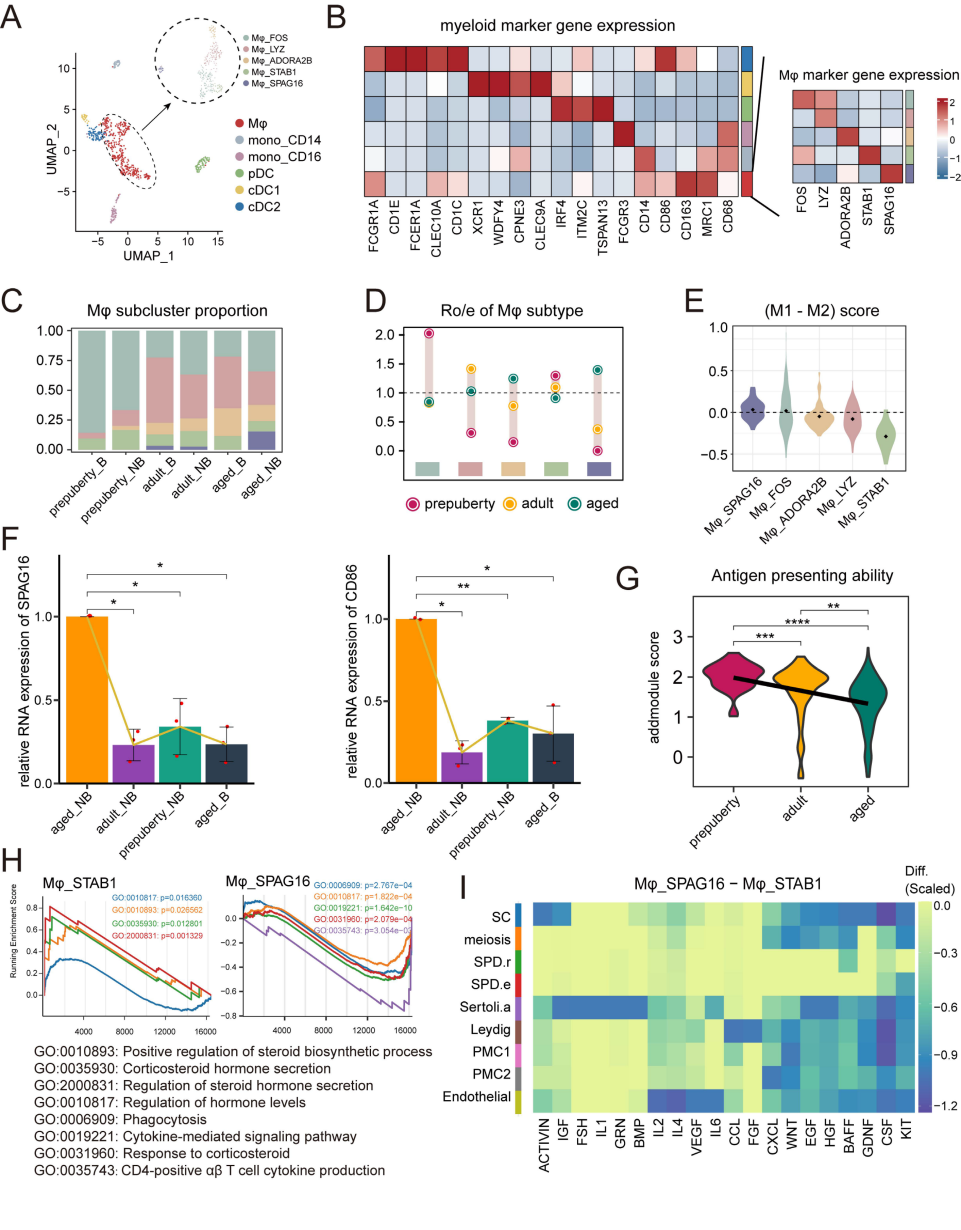
** Age-associated expansion of M1-like macrophages and reduced antigen-presentation signatures in the rhesus testis. A** UMAP plot illustrating subtypes of myeloid cells, with macrophage subpopulations highlighted within the dashed circle. **B** Heatmap plot showing expression patterns of specific marker genes for Myeloid subtypes and Macrophage subtypes. **C** Bar plot displaying proportional distribution of macrophage subtypes across age and seasonal conditions. **D** Plot displaying Ro/e scores of macrophage subtypes across age stages. **E** Assessment of M1 and M2 scores in macrophage subtypes by* Add modulescore function*. Values above the dotted line indicate a M1-biased state, while values below indicate a M2-biased state. **F** qPCR validation of representative markers (aged relative to adult; n = 3). **G** Violin plots depicting antigen presentation capacity (MHC class II module score) of macrophages across age stages. **H** GSEA showing functional pathway enrichment in *Mφ_STAB1* and *Mφ_SPAG16* macrophage subpopulations between breeding and non-breeding seasons. **I** Heatmap displays differential interaction effects of *Mφ_SPAG16* versus *Mφ_STAB1* on germ and somatic cell types. Negative values indicate weaker inferred effects for *Mφ_SPAG16* relative to *Mφ_STAB1*.

Macrophages represent the predominant immune population within the testicular interstitium, and their age-related alterations critically influence testicular homeostasis. Refined clustering of myeloid cells identified two functionally polarized macrophage subsets: Mφ_*SPAG16* subtype, exhibiting an M1-like pro-inflammatory signature and enriched in aged testes, and Mφ_*STAB1* subtype, exhibiting an M2-like anti-inflammatory signature and predominating in prepubertal and adult testes (Fig. 5A-E). Subsequent qPCR quantification further validated age-associated upregulation of Mφ_*SPAG16* markers (Fig. 5F). Notably, macrophages from aged testes exhibited reduced MHC class II antigen presentation capacity (Fig. 5G). Consistent with their polarization states, Mφ_*STAB1* macrophages were enriched for pathways involved in hormone levels, positive regulation of steroid biosynthetic process, corticosteroid hormone secretion, and regulation of steroid hormone secretion, whereas Mφ_*SPAG16* macrophages exhibited reduced signatures of phagocytosis, regulation of hormone levels, cytokine-mediated signaling pathway, response to corticosteroid, and CD4-positive aβ T cell cytokine production (Fig. 5H).

To assess how macrophage polarization may affect testicular function, we examined the age- and season-associated increases in Mφ_*SPAG16* macrophages and their interactions with somatic and germ cells (Fig. 5I). Cell-cell interaction analysis suggested that Mφ_SPAG16 displays altered communication patterns with somatic and germ-cell compartments during aging: (1) by interacting with testicular cells via CSF-related signaling, thereby reducing steroid production 35; (2) by disrupting FSH signaling through interactions with Sertoli cells, leading to reduced androgen levels^36^; and (3) by suppressing ACTIVIN/KIT pathways in SSCs, potentially disrupting spermatogenesis^20^. Collectively, these interaction landscapes position macrophages as integral components of the testicular immune microenvironment, coordinating immune modulation, endocrine regulation, and germ cell-associated signaling dynamics during aging and seasonal transitions.

## Discussion

Testicular aging disrupts germ cell function and androgen secretion, leading to fertility decline and systemic physiological dysfunction^37^. As ideal experimental models with high physiological and genetic similarity to humans, non-human primates provide critical translational references for studies of testicular development and aging 38-40. Recent single-cell transcriptomic analyses have reported SSC depletion and Sertoli cell homeostasis imbalance during primate testicular aging^26^, and epigenetic profiling has identified key regulatory factors involved in stem cell homeostasis in adult rhesus monkey^41^. Cross-species single-cell RNA sequencing has further elucidated conserved pathways and species-specific features across the human, macaque, and mouse spermatogenesis^32^. However, how intrinsic aging and reproductive seasonality shape rhesus monkey testicular dynamics remain underexplored. In our study, we defined key cell types, gene networks, and age/season-associated transcriptional programs, offering a comprehensive resource to decipher developmental and degenerative mechanisms in seasonally regulated testes of rhesus monkeys.

Sertoli cells are essential for germ cell development and spermatogenesis, providing nutritional and mechanical support and maintaining the specialized seminiferous microenvironment. In rodents, the BTB dynamically disassembles to allow preleptotene spermatocytes to pass, and its dysfunction directly disrupts germ cell differentiation^11^, ^12^. In aged non-human primate, Sertoli cells show significant downregulation of tight junction-related gene sets, consistent with compromised BTB integrity. In this study, we observe prominent seasonal fluctuations in meiotic germ cell abundance in adult rhesus testes, suggesting that meiotic dynamics are linked to reproductive rhythmicity. Mechanistically, our data support that Sertoli cells coordinately regulate tight and adherens junction programs, and dynamically reshaping BTB-associated junctional states across seasons. Specifically, in the non-breeding season, Sertoli cells display enhanced junctional programs and ultrastructural features consistent with a more “sealed” barrier state, coinciding with reduced meiotic progression. This finding extends the conventional view of BTB regulation by highlighting BTB plasticity as a seasonally tuned layer associated with meiotic dynamics in a seasonal primate, beyond the typical focus on pathological barrier disruption.

Beyond Sertoli cells, Leydig cells secrete testosterone to support masculinization and spermatogenesis, while peritubular myoid cells (PMC) facilitate sperm transport and microenvironment modulation, collectively maintaining testicular function and spermatogenic homeostasis^42^, ^43^. Human aging studies have reported reduced Hedgehog pathway receptor expression in Leydig cells, accompanied by PMC hyperplasia with reduced contractility and aberrant basement membrane secretion—findings consistent with observations in murine models^2^, ^44^. In addition to age-related signatures shared with humans and rodents, our data also discovered the seasonal specificity of Leydig, Sertoli, and PMC in rhesus monkeys. For example, in rhesus monkey, Leydig-3 supports cellular homeostasis and responds to seasonal cues, while Leydig-4 coordinates endocrine-immune interactions, and PMC1-2 dynamically regulates hemodynamics for seasonal adaptation. This spatial and functional partitioning (seminiferous epithelium versus interstitial compartment) ensures integrated regulation of spermatogenesis across aging and breeding seasons.

Spermatogonial stem cells represent another key node of age and season dependent regulation. SSCs not only possess the potential for directed differentiation but also maintain the homeostasis of the germline stem cell pool through self-renewal. Clinically, SSC transplantation represents a promising strategy for fertility preservation in pediatric patients exposed to gonadotoxic treatments. With childhood cancer 5-year survival rates exceeding 80%‌^45^ and therapy-associated germ cell depletion contributing to later infertility^46^‌, establishing robust SSC expansion systems is an unmet need. However, SSC biology differs substantially across species. Rodent spermatogonial cell classifications (Asingle (As), Apaired (Apr), Aaligned (Aal), A1-4, Int, B) 47 do not map directly onto the categories of humans (Adark, Apale, B) and monkeys (Adark, Apale, B1-4)^48^. Despite progress in culturing human undifferentiated spermatogonia, long-term ex *vivo* SSC expansion remains challenging in humans and non-human primates. Our study supports the interpretation of U3 as a high-stemness undifferentiated spermatogonial state with distinct age- and season-related dynamic characteristics. The abundance of U3 shows no seasonal fluctuations before sexual maturity, is restricted to the non-breeding season in adulthood, and is entirely absent during senescence. Notably, U3 exhibits the highest stemness among all SSC subsets, and shows low transcriptional similarity to reported human and murine SSC states, supporting lineage specificity. Importantly, U3 is characterized by PDGF-associated signaling pathway that peaks in adult testes during non-breeding season and declines with age. Guided by this seasonal niche signature, and informed by prior studies showing that VEGF/PDGF-nanohydrogels can promote vascular maturation via PDGF-driven collagen/fibronectin secretion^49^, ^50^, we developed a 3D collagen-PDGF scaffold that mimic seminiferous tubule conditions to sustain rhesus SSC stemness. Using this system, we achieved enhanced clone formation and sustained proliferation beyond 14 days, together with transcriptional features consistent with increased stemness-associated activity. These findings provide a practical direction for improving primate SSC culture strategies and for modeling age-associated decline of the SSC pool. However, the precise mechanistic role of PDGF signaling remains to be further validated via in vivo functional investigations.

In addition, our data highlight immune compositional changes as a prominent feature of testicular aging. Consistent with the concept that immune homeostasis is intertwined with endocrine and spermatogenic function^51^, ^52^, we observe shifts in immune composition and macrophage polarization with age, together suggesting an increasingly pro-inflammatory niche context. In aged rhesus testes, we observed a marked enrichment of CD3⁺CD4⁻CD8⁻ T cells, a subset with poorly defined roles in the gonadal niche. Pathway enrichment analysis suggested that aging was associated with reduced innate and regulatory immune responses and diminished T cell functional signatures, suggesting a potential weakening of immune surveillance in the aged testicular niche. These findings raise the possibility that CD3⁺CD4⁻CD8⁻ T cells may represent a previously underappreciated layer of immune regulation in the primate testis. However, additional validation and functional studies will be required to define their identity, localization, and mechanistic relevance.

Concurrently, testicular macrophages, the predominant immune population in the interstitium, shift toward a pro-inflammatory phenotype with aging. This is accompanied by diminished antigen presentation signatures and impaired apoptotic cell clearance, thereby sustaining chronic inflammation and compromising local immune privilege. Such changes not only exacerbate age-related tissue degeneration but also weaken macrophage support for germ cell development. Previous study has shown that M-CSF-deficient mice exhibit infertility and oligospermia^53^, though it remains unclear whether this reflects direct effects on SSCs or indirect regulation via macrophage-somatic cell interactions. In line with this, our results revealed particularly robust SSC-macrophage interactions compared to SSC-somatic cell signaling, suggesting that macrophages regulate SSC maintenance via M-CSF mediated niche-specific crosstalk, and age-associated immune dysfunction may disrupt this axis, contributing to progressive germ cell loss.

Despite the depth of our single-cell atlas, several limitations should be noted. First, this study included a limited number of rhesus monkeys, and larger cohorts will be needed to further strengthen the generalizability of our findings. Although the paired seasonal sampling design reduced inter-individual variability, physiological compensation following unilateral orchiectomy and non-seasonal temporal variation cannot be fully ruled out. Finally, although our transcriptomic and computational analyses provide important clues to potential regulatory mechanisms, the roles of PDGF-associated signaling, CD3^+^CD4^-^CD8^-^ T-cell expansion, and macrophage polarization in SSC maintenance, spermatogenesis, and steroidogenesis remain to be functionally validated in future studies.

In conclusion, this work establishes the first single-cell resource for the rhesus macaque testis across age stages and reproductive seasons, highlighting season-associated regulation of meiosis and SSC heterogeneity together with aging-related changes of somatic support and immune microenvironments. First, Sertoli cells exhibit season-associated remodeling of tight-junction programs that coincides with changes in meiotic germ-cell states. Second, we identify a transcriptionally distinct, high-stemness undifferentiated spermatogonial state (U3) that is selectively enriched during the adult non-breeding season and progressively depleted with aging. Third, testicular aging is accompanied by immune changes, including pro-inflammatory macrophage polarization and shifts in T-cell composition, together consistent with heightened inflammatory pressure on spermatogenic homeostasis. These findings advance our understanding of how aging and seasonal transitions shape primate testicular cellular hierarchies and niche homeostasis, while providing a valuable single-cell resource for future functional studies.

## Supplementary Material

Supplementary figures and tables.

## Data Availability

The ScRNA-seq and bulk RNA-seq raw data generated in this study have been deposited in the GEO Omnibus database under accession code GSE306699 and GSE306702 respectively. Processed scRNA-seq and bulk RNA-seq data are also available through these GEO accession records. Source data are provided with this paper.

## References

[1] Guo J, Sosa E, Chitiashvili T, Nie X, Rojas EJ, Oliver E (2021). Single-cell analysis of the developing human testis reveals somatic niche cell specification and fetal germline stem cell establishment. Cell stem cell.

[2] Nie X, Munyoki SK, Sukhwani M, Schmid N, Missel A, Emery BR (2022). Single-cell analysis of human testis aging and correlation with elevated body mass index. Developmental cell.

[3] Guo J, Nie X, Giebler M, Mlcochova H, Wang Y, Grow EJ (2020). The Dynamic Transcriptional Cell Atlas of Testis Development during Human Puberty. Cell stem cell.

[4] Cui L, Nie X, Guo Y, Ren P, Guo Y, Wang X (2025). Single-cell transcriptomic atlas of the human testis across the reproductive lifespan. Nature aging.

[5] Uno H (1997). Age-related pathology and biosenescent markers in captive rhesus macaques. Age.

[6] Wickings EJ, Nieschlag E (1980). Seasonality in endocrine and exocrine testicular function of the adult rhesus monkey (Macaca mulatta) maintained in a controlled laboratory environment. International journal of andrology.

[7] Farbehi N, Patrick R, Dorison A, Xaymardan M, Janbandhu V, Wystub-Lis K (2019). Single-cell expression profiling reveals dynamic flux of cardiac stromal, vascular and immune cells in health and injury. eLife.

[8] Dadhich RK, Barrionuevo FJ, Real FM, Lupiañez DG, Ortega E, Burgos M (2013). Identification of live germ-cell desquamation as a major mechanism of seasonal testis regression in mammals: a study in the Iberian mole (Talpa occidentalis). Biology of reproduction.

[9] Jiménez R, Burgos M, Barrionuevo FJ (2015). Circannual Testis Changes in Seasonally Breeding Mammals. Sexual development: genetics, molecular biology, evolution, endocrinology, embryology, and pathology of sex determination and differentiation.

[10] You X, Chen Q, Yuan D, Zhang C, Zhao H (2021). Common markers of testicular Sertoli cells. Expert review of molecular diagnostics.

[11] Mruk DD, Cheng CY (2004). Sertoli-Sertoli and Sertoli-germ cell interactions and their significance in germ cell movement in the seminiferous epithelium during spermatogenesis. Endocrine reviews.

[12] Cheng CY, Mruk DD (2002). Cell junction dynamics in the testis: Sertoli-germ cell interactions and male contraceptive development. Physiological reviews.

[13] Wu S, Wang L, Tang EI, Wang J, Cheng CY (2021). An In Vitro Assay to Monitor Sertoli Cell Blood-Testis Barrier (BTB) Integrity. Methods in molecular biology (Clifton, NJ).

[14] Wong CH, Cheng CY (2005). The blood-testis barrier: its biology, regulation, and physiological role in spermatogenesis. Current topics in developmental biology.

[15] Shima Y, Miyabayashi K, Haraguchi S, Arakawa T, Otake H, Baba T (2013). Contribution of Leydig and Sertoli cells to testosterone production in mouse fetal testes. Molecular endocrinology (Baltimore, Md).

[16] Haywood M, Spaliviero J, Jimemez M, King NJ, Handelsman DJ, Allan CM (2003). Sertoli and germ cell development in hypogonadal (hpg) mice expressing transgenic follicle-stimulating hormone alone or in combination with testosterone. Endocrinology.

[17] Wang M, Liu X, Chang G, Chen Y, An G, Yan L (2018). Single-Cell RNA Sequencing Analysis Reveals Sequential Cell Fate Transition during Human Spermatogenesis. Cell stem cell.

[18] Li SY, Kumar S, Gu X, DeFalco T (2024). Testicular immunity. Molecular aspects of medicine.

[19] Zhao S, Zhu W, Xue S, Han D (2014). Testicular defense systems: immune privilege and innate immunity. Cellular & molecular immunology.

[20] Meinhardt A, Dejucq-Rainsford N, Bhushan S (2022). Testicular macrophages: development and function in health and disease. Trends in immunology.

[21] Walker ML, Wilson ME, Gordon TP (1984). Endocrine control of the seasonal occurrence of ovulation in rhesus monkeys housed outdoors. Endocrinology.

[22] Ramaswamy S, Marshall GR, McNeilly AS, Plant TM (2000). Dynamics of the follicle-stimulating hormone (FSH)-inhibin B feedback loop and its role in regulating spermatogenesis in the adult male rhesus monkey (Macaca mulatta) as revealed by unilateral orchidectomy. Endocrinology.

[23] Liu Y, Sanoff HK, Cho H, Burd CE, Torrice C, Ibrahim JG (2009). Expression of p16(INK4a) in peripheral blood T-cells is a biomarker of human aging. Aging cell.

[24] Xin-Chang Z, Peng W, Zhao-Yuan H, Xiao-Bin H, Ru-Jin Z, Yi-Xun L (2002). Expression of P16(INK4a) in testis of rhesus monkey during heat stress and testosterone undecanoate induced azoospermia or oligozoospermia. Contraception.

[25] Paniagua R, Amat P, Nistal M, Martin A (1986). Ultrastructure of Leydig cells in human ageing testes. Journal of anatomy.

[26] Huang D, Zuo Y, Zhang C, Sun G, Jing Y, Lei J (2023). A single-nucleus transcriptomic atlas of primate testicular aging reveals exhaustion of the spermatogonial stem cell reservoir and loss of Sertoli cell homeostasis. Protein & cell.

[27] Lau X, Munusamy P, Ng MJ, Sangrithi M (2020). Single-Cell RNA Sequencing of the Cynomolgus Macaque Testis Reveals Conserved Transcriptional Profiles during Mammalian Spermatogenesis. Developmental cell.

[28] Krasic J, Skara Abramovic L, Himelreich Peric M, Vanjorek V, Gangur M, Zovko D (2023). Testicular Germ Cell Tumor Tissue Biomarker Analysis: A Comparison of Human Protein Atlas and Individual Testicular Germ Cell Tumor Component Immunohistochemistry. Cells.

[29] Su J, Yang Y, Wang D, Su H, Zhao F, Zhang C (2025). A dynamic transcriptional cell atlas of testes development after birth in Hu sheep. BMC biology.

[30] Moon C, Jeong CW, Kim H, Ahn M, Kim S, Shin T (2006). Expression of CD44 adhesion molecule in rat testis with ischemia/reperfusion injury. The Journal of veterinary medical science.

[31] Chen SR, Liu YX (2015). Regulation of spermatogonial stem cell self-renewal and spermatocyte meiosis by Sertoli cell signaling. Reproduction (Cambridge, England).

[32] Shami AN, Zheng X, Munyoki SK, Ma Q, Manske GL, Green CD (2020). Single-Cell RNA Sequencing of Human, Macaque, and Mouse Testes Uncovers Conserved and Divergent Features of Mammalian Spermatogenesis. Developmental cell.

[33] Green CD, Ma Q, Manske GL, Shami AN, Zheng X, Marini S (2018). A Comprehensive Roadmap of Murine Spermatogenesis Defined by Single-Cell RNA-Seq. Developmental cell.

[34] Achita P, Dervovic D, Ly D, Lee JB, Haug T, Joe B (2018). Infusion of ex-vivo expanded human TCR-αβ(+) double-negative regulatory T cells delays onset of xenogeneic graft-versus-host disease. Clinical and experimental immunology.

[35] Jorban A, Lunenfeld E, Huleihel M (2023). Granulocyte-macrophage colony-stimulating factor (GM-CSF)-induced maturation of spermatogonial cells from prepubertal mice in vitro is enhanced by testosterone. European cytokine network.

[36] Lei ZM, Mishra S, Ponnuru P, Li X, Yang ZW, Rao Ch V (2004). Testicular phenotype in luteinizing hormone receptor knockout animals and the effect of testosterone replacement therapy. Biology of reproduction.

[37] Decaroli MC, Rochira V (2017). Aging and sex hormones in males. Virulence.

[38] Fayomi AP, Orwig KE (2018). Spermatogonial stem cells and spermatogenesis in mice, monkeys and men. Stem cell research.

[39] Fang X, Jiang M, Zhou M, Shao J, Fang X, Wang J (2022). Elucidating the developmental dynamics of mouse stromal cells at single-cell level. Life medicine.

[40] Zou X, Dai X, Mentis AA, Esteban MA, Liu L, Han L (2022). From monkey single-cell atlases into a broader biomedical perspective. Life medicine.

[41] Bi R, Pan LN, Dai H, Sun C, Li C, Lin HJ (2024). Epigenetic characterization of adult rhesus monkey spermatogonial stem cells identifies key regulators of stem cell homeostasis. Nucleic acids research.

[42] Mäkelä JA, Koskenniemi JJ, Virtanen HE, Toppari J (2019). Testis Development. Endocrine reviews.

[43] Chen P, Zirkin BR, Chen H (2020). Stem Leydig Cells in the Adult Testis: Characterization, Regulation and Potential Applications. Endocrine reviews.

[44] Garcia TX, Farmaha JK, Kow S, Hofmann MC (2014). RBPJ in mouse Sertoli cells is required for proper regulation of the testis stem cell niche. Development (Cambridge, England).

[45] Winther JF, Kenborg L, Byrne J, Hjorth L, Kaatsch P, Kremer LC (2015). Childhood cancer survivor cohorts in Europe. Acta oncologica (Stockholm, Sweden).

[46] Masliukaite I, Ntemou E, Feijen EAM, van de Wetering M, Meissner A, Soufan AT (2023). Childhood cancer and hematological disorders negatively affect spermatogonial quantity at diagnosis: a retrospective study of a male fertility preservation cohort. Human reproduction (Oxford, England).

[47] de Rooij DG (1973). Spermatogonial stem cell renewal in the mouse. I. Normal situation. Cell and tissue kinetics.

[48] Clermont Y (1972). Kinetics of spermatogenesis in mammals: seminiferous epithelium cycle and spermatogonial renewal. Physiological reviews.

[49] Skinner MK (1991). Cell-cell interactions in the testis. Endocrine reviews.

[50] Bellvé AR, Zheng W (1989). Growth factors as autocrine and paracrine modulators of male gonadal functions. Journal of reproduction and fertility.

[51] Xia K, Luo P, Yu J, He S, Dong L, Gao F (2024). Single-cell RNA sequencing reveals transcriptomic landscape and potential targets for human testicular ageing. Human reproduction (Oxford, England).

[52] Bhushan S, Theas MS, Guazzone VA, Jacobo P, Wang M, Fijak M (2020). Immune Cell Subtypes and Their Function in the Testis. Frontiers in immunology.

[53] Cohen PE, Chisholm O, Arceci RJ, Stanley ER, Pollard JW (1996). Absence of colony-stimulating factor-1 in osteopetrotic (csfmop/csfmop) mice results in male fertility defects. Biology of reproduction.

