# Supplementary material for: Single-cell atlas of rhesus monkey testis reveals aging- and season-dependent regulation of spermatogenesis and microenvironment homeostasis
Source: Int J Biol Sci. 2026 Jul 20;22(12):6858–75. doi: 10.7150/ijbs.134245 (PMC13412284; doi:10.7150/ijbs.134245)
Supplement: Supplementary file 1 — Supplementary figures and tables. [file ijbsv22p6858s1.pdf]

Figure S1. Aging-associated histological alterations in rhesus monkey testes. Immunofluorescence staining of P16 (senescence marker), and Collagen IV (marker of basal membrane of seminiferous tubules). Left panels: representative images. Right panels: quantification of P16-positive cells, as well as basement membrane (BM) thickness based on collagen IV staining, presented as fold changes (mean ± SEM). Scale bars, 20 μm for P16 and 50 μm for collagen IV staining.

Figure S2. Pseudotime analysis of germ cell development across age and seasons
A Pseudotime analysis depicting germ cell development from SCto SPD.e across age and seasonal conditions. **B** Heatmaps and curve charts showing the dynamic expression profiles of genes along the pseudotime trajectory of meiosis. The heatmap provides a visual representation of the expression levels of these prominent DEGs. Line plots illustrate gene expression trends, and bar charts summarize the enrichment of associated GO terms for each gene cluster.

Figure S3. Somatic cell subtypes and functional programs in the rhesus testis across age and reproductive seasons
A UMAP plot showing subtypes of Sertoli cells. **B** Ro/e value indicates the enrichment levels of each Sertoli cell subtype across age/seasonal conditions. **C** UMAP plots showing the expression pattern of SOX9 and AMH. **D** Lollipop plot displaying significantly enriched upregulated (right) and downregulated (left) pathways in sertoli.a cells compared with sertoli.p. **E** Lollipop plot showing enriched pathways in adult Sertoli.a cells (breeding versus non-breeding seasons). **F** UMAP plot illustrating subtypes of Leydig cells. **G** Ro/e value indicates the enrichment levels of each Leydig cell subtype across different age/seasonal conditions. **H** Dotplot illustrating the distinct expression patterns of subtype-specific marker genes across Leydig cell populations. **I** Lollipop plot displays significantly enriched pathways in adult Leydig-3 cells (breeding versus non-breeding seasons). **J** Lollipop plots illustrating specifically enriched upregulated (right) and downregulated (left) pathways in Leydig-1&2, Leydig-3, and leydig-4 cells. **K** UMAP plot illustrating subtypes of PMC cells. **L** Ro/e value indicates the enrichment levels of PMC cell subtypes across different age/seasonal conditions. **M** Dotplot illustrating the distinct expression patterns of subtype-specific marker genes across PMC cell populations. **N** Lollipop plot displaying significantly downregulated pathways in PMC1_1 cells in aged monkeys compared with prepuberty and adult

groups. **O** Lollipop plot displaying significantly upregulated pathways in adult PMC1_2 cells (breeding versus non-breeding seasons).

Figure S4. Gene expression and intercellular communication among immune, germ, and somatic cells across age and seasonal conditions

A Interaction strength among immune cells, germ cells, and somatic cells under different age/seasonal conditions. **B** Chord diagram showing directional ligand (immune cells)-receptor (germ cells) interactions across adult breeding, adult non-breeding, aged breeding and aged non-breeding seasons. **C** Chord diagram visualizes directional ligand (immune cells)-receptor (somatic cells) interactions across adult breeding, adult non-breeding, aged breeding, and aged non-breeding seasons. **D** Chord diagram visualizes directional ligand (somatic cells)-receptor (germ cells) interactions across adult breeding, adult non-breeding, aged breeding, and aged non-breeding seasons.

Figure S5. U3 definition, regulatory programs, and cross-species comparison

A Circular plot showing Augur algorithm-based ranking of spermatogenic cell types by responsiveness to age and seasonal effects. **B** UMAP visualization showing the distribution of SSC subpopulations (U1–U3, dif, end) across age and seasonal conditions. **C** Bar plots showing the counts of enriched GO terms for U3 versus u1/u2 in adult testes during the non-breeding season. **D** Heatmap showing regulon activity across SC subpopulations. Regulon activity was scaled by row (z-score) and grouped into five modules (c1–c5) using hierarchical clustering (complete linkage). For each module, representative enriched GO terms of regulon target genes are shown. Dot plots display enrichment significance (−log10 *P* value), and bar plots indicate the corresponding gene counts for each GO term. **E** UMAP visualization showing distribution of SSC subpopulations in mouse and human. Dot plots showing expression patterns of substype-specific marker genes. **F** Cross-species gene expression correlation analysis of SSC subpopulations, comparing rhesus U3 with reported human and mouse SSC states.

Fig.S6 Age-Associated expansion and functional attenuation of DNT cells in the rhesus testis

A UMAP plot depicting the distribution of T cell populations. **B** Bar plot with nested percentage information showing T cell subpopulation composition across age and seasonal conditions. Percentages shown at the right of each bar indicate the proportion of T cells among total cells for each condition. Percentages within the stacked bars (and pie chart) indicate the relative proportions of CD4, CD8, and DNT subsets within the T cell compartment. The pie chart summarizes overall T cell subtype composition. **C** Representative flow cytometry plots for *CD3* expression in testicular cells within each condition. **D** qPCR analysis of *CD3D* expression in adult testes relative to aged group in breeding or nonbreeding seasons. **E** Lollipop plot displaying showing pathways downregulated in DNT cells in aged versus prepubertal/adult testes.

Figure S1

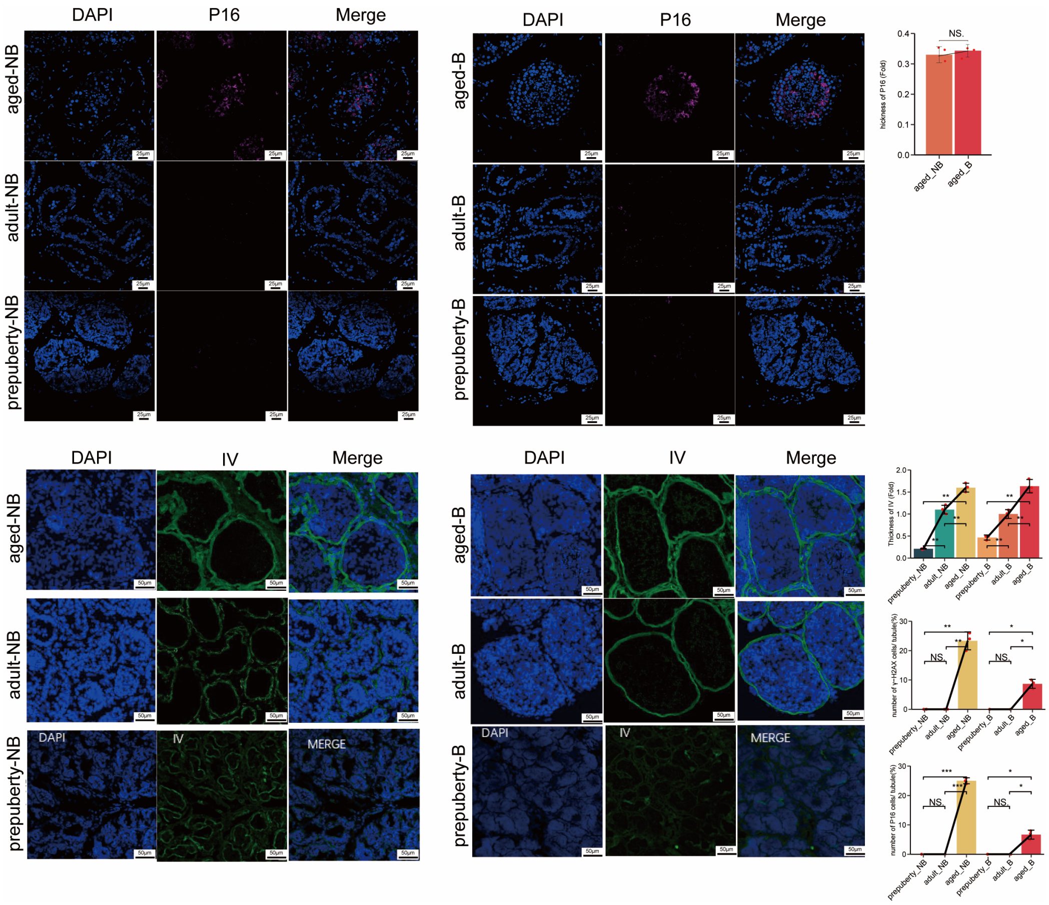

DAPI
P16
Merge
aged-NB
adult-NB
prepuberty-NB
aged-B
adult-B
prepuberty-B
NS.
aged_NB
aged_B
IV
MERGE
prepuberty_NB
adult_NB
prepuberty_B
adult_B
Thickness of IV (Fold)
NS

Figure S2

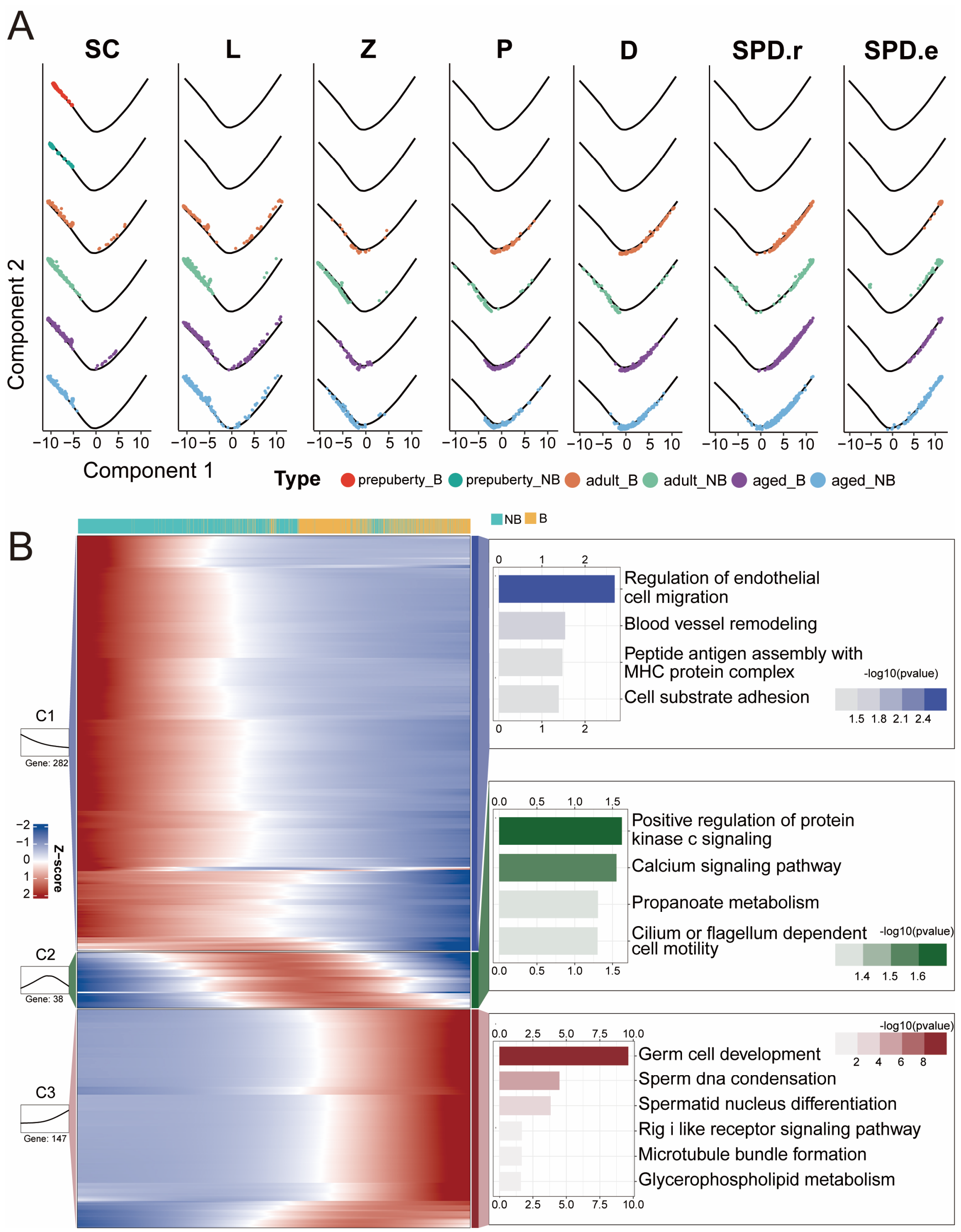

A
SC
L
Z
P
D
SPD.r
SPD.e
Component 2
Component 1
Type
prepuberty_B
prepuberty_NB
adult_B
adult_NB
aged_B
aged_NB
B
NB
B
C1
Gene: 282
C2
Gene: 38
C3
Gene: 147
Z-score
Regulation of endothelial cell migration
Blood vessel remodeling
Peptide antigen assembly with MHC protein complex
Cell substrate adhesion
-log10(pvalue)
Positive regulation of protein kinase c signaling
Calcium signaling pathway
Propanoate metabolism
Cilium or flagellum dependent cell motility
Germ cell development
Sperm dna condensation
Spermatid nucleus differentiation
Rig i like receptor signaling pathway
Microtubule bundle formation
Glycerophospholipid metabolism

Figure S3

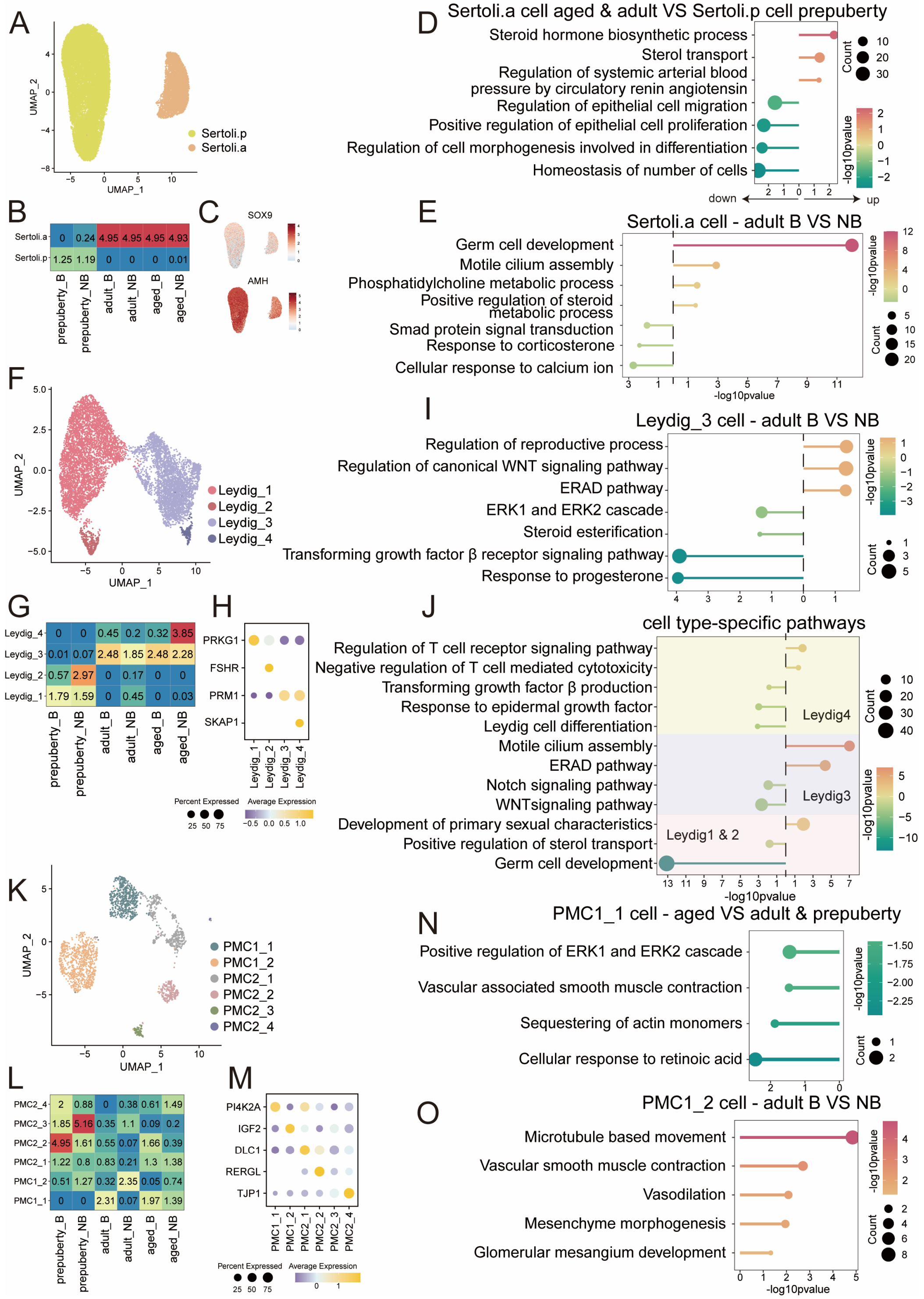

A
UMAP_2
UMAP_1
Sertoli.p
Sertoli.a
B
Sertoli.a 0 0.24 4.95 4.95 4.95 4.93
Sertoli.p 1.25 1.19 0 0 0 0.01
prepuberty_B prepuberty_NB adult_B adult_NB aged_B aged_NB
C
SOX9
AMH
D
Sertoli.a cell aged & adult VS Sertoli.p cell prepuberty
Steroid hormone biosynthetic process
Sterol transport
Regulation of systemic arterial blood pressure by circulatory renin angiotensin
Regulation of epithelial cell migration
Positive regulation of epithelial cell proliferation
Regulation of cell morphogenesis involved in differentiation
Homeostasis of number of cells
Count
-log10pvalue
down
up
E
Sertoli.a cell - adult B VS NB
Germ cell development
Motile cilium assembly
Phosphatidylcholine metabolic process
Positive regulation of steroid metabolic process
Smad protein signal transduction
Response to corticosterone
Cellular response to calcium ion
-log10pvalue
Count
F
UMAP_2
UMAP_1
Leydig_1
Leydig_2
Leydig_3
Leydig_4
I
Leydig_3 cell - adult B VS NB
Regulation of reproductive process
Regulation of canonical WNT signaling pathway
ERAD pathway
ERK1 and ERK2 cascade
Steroid esterification
Transforming growth factor β receptor signaling pathway
Response to progesterone
-log10pvalue
Count
G
Leydig_4 0 0 0.45 0.2 0.32 3.85
Leydig_3 0.01 0.07 2.48 1.85 2.48 2.28
Leydig_2 0.57 2.97 0 0.17 0 0
Leydig_1 1.79 1.59 0 0.45 0 0.03
prepuberty_B prepuberty_NB adult_B adult_NB aged_B aged_NB
H
PRKG1
FSHR
PRM1
SKAP1
Leydig_1 Leydig_2 Leydig_3 Leydig_4
Percent Expressed
Average Expression
J
cell type-specific pathways
Regulation of T cell receptor signaling pathway
Negative regulation of T cell mediated cytotoxicity
Transforming growth factor β production
Response to epidermal growth factor
Leydig cell differentiation
Motile cilium assembly
ERAD pathway
Notch signaling pathway
WNTsignaling pathway
Development of primary sexual characteristics
Positive regulation of sterol transport
Germ cell development
Leydig4
Leydig3
Leydig1 & 2
Count
-log10pvalue
K
UMAP_2
UMAP_1
PMC1_1
PMC1_2
PMC2_1
PMC2_2
PMC2_3
PMC2_4
N
PMC1_1 cell - aged VS adult & prepuberty
Positive regulation of ERK1 and ERK2 cascade
Vascular associated smooth muscle contraction
Sequestering of actin monomers
Cellular response to retinoic acid
-log10pvalue
Count
L
PMC2_4 2 0.88 0 0.38 0.61 1.49
PMC2_3 1.85 5.16 0.35 1.1 0.09 0.2
PMC2_2 4.95 1.61 0.55 0.07 1.66 0.39
PMC2_1 1.22 0.8 0.83 0.21 1.3 1.38
PMC1_2 0.51 1.27 0.32 2.35 0.05 0.74
PMC1_1 0 0 2.31 0.07 1.97 1.39
prepuberty_B prepuberty_NB adult_B adult_NB aged_B aged_NB
M
PI4K2A
IGF2
DLC1
RERGL
TJP1
PMC1_1 PMC1_2 PMC2_1 PMC2_2 PMC2_3 PMC2_4
Percent Expressed
Average Expression
O
PMC1_2 cell - adult B VS NB
Microtubule based movement
Vascular smooth muscle contraction
Vasodilation
Mesenchyme morphogenesis
Glomerular mesangium development
-log10pvalue
Count

Figure S4

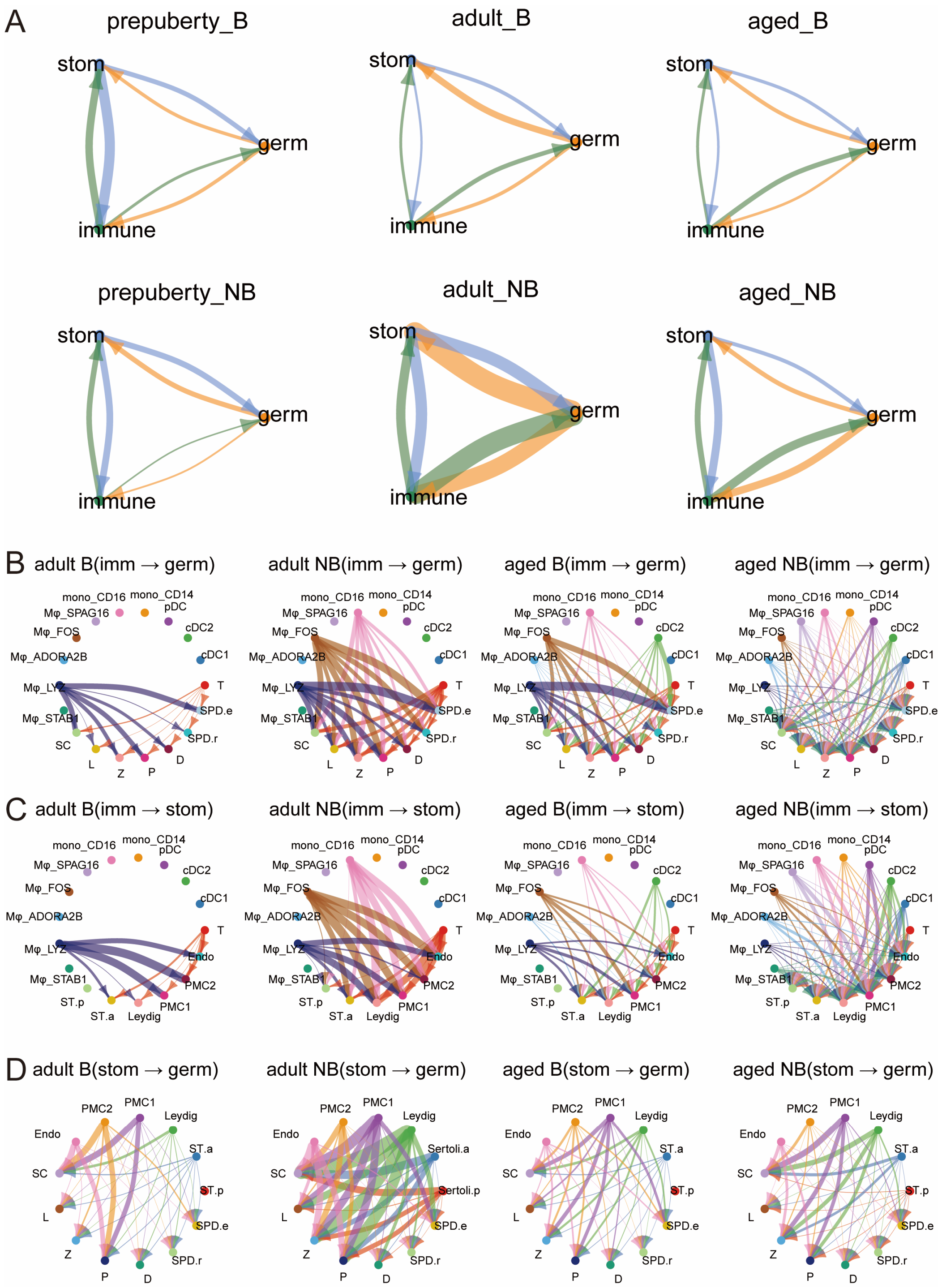
A
prepuberty_B
adult_B
aged_B
prepuberty_NB
adult_NB
aged_NB
stom
germ
immune
B adult B(imm → germ)
adult NB(imm → germ)
aged B(imm → germ)
aged NB(imm → germ)
mono_CD16
mono_CD14
pDC
Mφ_SPAG16
Mφ_FOS
cDC2
cDC1
Mφ_ADORA2B
Mφ_LYZ
T
SPD.e
Mφ_STAB1
SPD.r
SC
L
Z
P
D
C adult B(imm → stom)
adult NB(imm → stom)
aged B(imm → stom)
aged NB(imm → stom)
Endo
PMC2
PMC1
ST.p
ST.a
Leydig
D adult B(stom → germ)
adult NB(stom → germ)
aged B(stom → germ)
aged NB(stom → germ)
ST.p
Sertoli.a
Sertoli.p

Figure S5

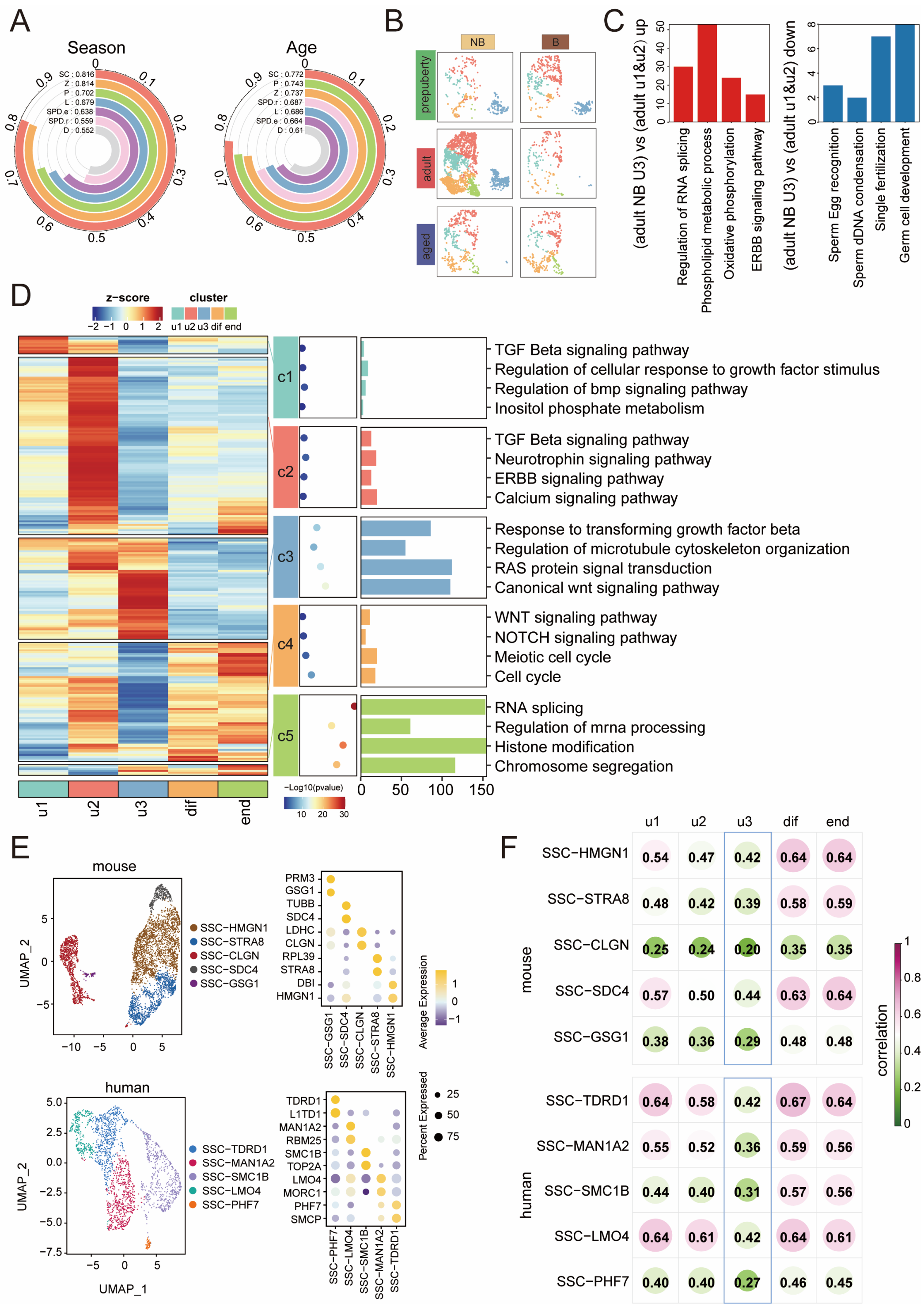
A
Season
Age
SC : 0.816
Z : 0.814
P : 0.702
L : 0.679
SPD.e : 0.638
SPD.r : 0.559
D : 0.552
SC : 0.772
P : 0.743
Z : 0.737
SPD.r : 0.687
L : 0.686
SPD.e : 0.664
D : 0.61
B
NB
B
prepuberty
adult
aged
C
(adult NB U3) vs (adult u1&u2) up
(adult NB U3) vs (adult u1&u2) down
Regulation of RNA splicing
Phospholipid metabolic process
Oxidative phosphorylation
ERBB signaling pathway
Sperm Egg recognition
Sperm dDNA condensation
Single fertilization
Germ cell development
D
z-score
cluster
u1 u2 u3 dif end
c1
c2
c3
c4
c5
TGF Beta signaling pathway
Regulation of cellular response to growth factor stimulus
Regulation of bmp signaling pathway
Inositol phosphate metabolism
TGF Beta signaling pathway
Neurotrophin signaling pathway
ERBB signaling pathway
Calcium signaling pathway
Response to transforming growth factor beta
Regulation of microtubule cytoskeleton organization
RAS protein signal transduction
Canonical wnt signaling pathway
WNT signaling pathway
NOTCH signaling pathway
Meiotic cell cycle
Cell cycle
RNA splicing
Regulation of mrna processing
Histone modification
Chromosome segregation
−Log10(pvalue)
u1
u2
u3
dif
end
E
mouse
SSC−HMGN1
SSC−STRA8
SSC−CLGN
SSC−SDC4
SSC−GSG1
human
SSC−TDRD1
SSC−MAN1A2
SSC−SMC1B
SSC−LMO4
SSC−PHF7
UMAP_1
UMAP_2
Average Expression
Percent Expressed
F
u1
u2
u3
dif
end
mouse
human
correlation
SSC−HMGN1 0.54 0.47 0.42 0.64 0.64
SSC−STRA8 0.48 0.42 0.39 0.58 0.59
SSC−CLGN 0.25 0.24 0.20 0.35 0.35
SSC−SDC4 0.57 0.50 0.44 0.63 0.64
SSC−GSG1 0.38 0.36 0.29 0.48 0.48
SSC−TDRD1 0.64 0.58 0.42 0.67 0.64
SSC−MAN1A2 0.55 0.52 0.36 0.59 0.56
SSC−SMC1B 0.44 0.40 0.31 0.57 0.56
SSC−LMO4 0.64 0.61 0.42 0.64 0.61
SSC−PHF7 0.40 0.40 0.27 0.46 0.45

Figure S6

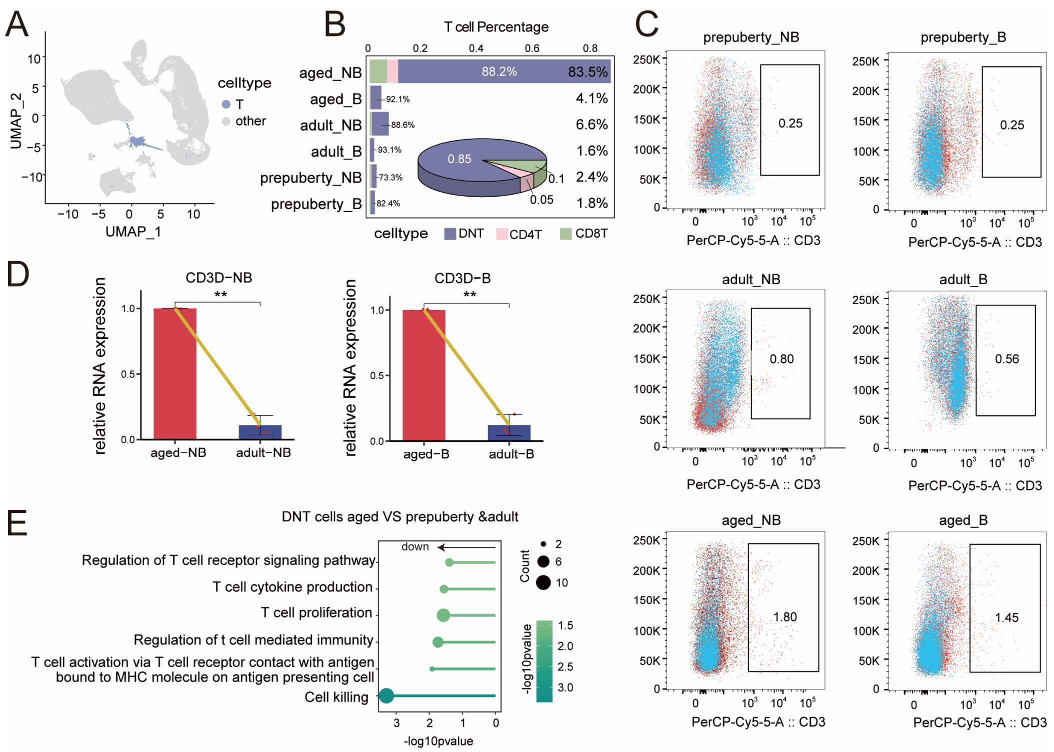

A
celltype
T
other
UMAP_1
UMAP_2
B
T cell Percentage
aged_NB
aged_B
adult_NB
adult_B
prepuberty_NB
prepuberty_B
88.2%
83.5%
4.1%
6.6%
1.6%
2.4%
1.8%
92.1%
88.6%
93.1%
73.3%
82.4%
0.85
0.1
0.05
celltype
DNT
CD4T
CD8T
C
prepuberty_NB
prepuberty_B
adult_NB
adult_B
aged_NB
aged_B
0.25
0.25
0.80
0.56
1.80
1.45
PerCP-Cy5-5-A :: CD3
D
CD3D-NB
CD3D-B
relative RNA expression
aged-NB
adult-NB
aged-B
adult-B
**
E
DNT cells aged VS prepuberty &adult
down
Regulation of T cell receptor signaling pathway
T cell cytokine production
T cell proliferation
Regulation of t cell mediated immunity
T cell activation via T cell receptor contact with antigen bound to MHC molecule on antigen presenting cell
Cell killing
Count
-log10pvalue

Table S1: PCR primer sequences

Name of primer	primer sequence
APOA1-F	5’-AAGATGAACCCCCACAGACC-3’
APOA1-R	5’-GGACACATAGTCTTTGCCGC-3’
VIM-F	5’-GGACCAGCTAACCAACGACA-3’
VIM-R	5’-AGATTGCAGGGTGTTCTCGG-3’
PPM1E-F	5’-GCCTGCAGCAGCTCTACAAATATAA-3’
PPM1E-R	5’-AGTCCCTTCTGTGCCATCCG-3’
PRSS21-F	5’-TCAGAACCGGACAGACTGCT-3’
PRSS21-R	5’-TCCTGGAGGGTATAGGGAGATG-3’
CD3D-F	5’-GCTACCCTTCTCTCCCAAGTG-3’
CD3D-R	5’-ACCGTTCCCTCTACCCATGT-3’
CD86-F	5’-AAACCGAAGCCTGAGTGAGC-3’
CD86-R	5’-GTGCGGCCCATATACTTGGA-3’
SPAG16-F	5’-CGTTGGGCATGGGTTTGAC-3’
SPAG16-R	5’-CCACGGGAAGGATACGCTC-3’

Table S2. Animal and single-cell sequencing metadata

Sample ID	Age group	Sex	Season	Mean UMI Counts per Cell
RNA-195041	prepuberty	male	NB	3734.83
RNA-195041-12	prepuberty	male	B	4322.74
RNA-197007	prepuberty	male	NB	3850.52
RNA-197007-12	prepuberty	male	B	3803.62
RNA-145269	adult	male	NB	4243.52
RNA-145269-12	adult	male	B	3370.64
RNA-146057	adult	male	NB	3235.34
RNA-146057-12	adult	male	B	3850
RNA-98093	aged	male	NB	2464.58
RNA-98093-12	aged	male	B	2852.15
RNA-98333	aged	male	NB	2907.17
RNA-98333-12	aged	male	B	2769.66

Table S3. SSC- monkey_human_correlation

	95% CI Lower Limit	Correlation Coefficient	95% CI Upper Limit	P value	95% CI Summary
SSC-TDRD1-u1	0.63	0.64	0.65	0	0.64 (0.63-0.65)
SSC-MAN1A-u1	0.54	0.55	0.56	0	0.55 (0.54-0.56)
SSC-SMC1B-u1	0.42	0.44	0.45	0	0.44 (0.42-0.45)
SSC-LMO4-u1	0.63	0.64	0.65	0	0.64 (0.63-0.65)
SSC-PHF7-u1	0.39	0.4	0.42	0	0.4 (0.39-0.42)
SSC-TDRD1-u2	0.57	0.58	0.59	0	0.58 (0.57-0.59)
SSC-MAN1A-u2	0.5	0.52	0.53	0	0.52 (0.5-0.53)
SSC-SMC1B-u2	0.38	0.4	0.42	0	0.4 (0.38-0.42)
SSC-LMO4-u2	0.59	0.61	0.62	0	0.61 (0.59-0.62)
SSC-PHF7-u2	0.39	0.4	0.42	0	0.4 (0.39-0.42)
SSC-TDRD1-u3	0.4	0.42	0.43	0	0.42 (0.4-0.43)
SSC-MAN1A-u3	0.35	0.36	0.38	0	0.36 (0.35-0.38)
SSC-SMC1B-u3	0.3	0.31	0.33	9.29611844665874e-259	0.31 (0.3-0.33)

SSC-LMO4-u3	0.4	0.42	0.43	0	0.42 (0.4-0.43)
SSC-PHF7-u3	0.25	0.27	0.28	2.73278287417653e-187	0.27 (0.25-0.28)
SSC-TDRD1-dif	0.66	0.67	0.68	0	0.67 (0.66-0.68)
SSC-MAN1A-dif	0.58	0.59	0.6	0	0.59 (0.58-0.6)
SSC-SMC1B-dif	0.56	0.57	0.59	0	0.57 (0.56-0.59)
SSC-LMO4-dif	0.63	0.64	0.65	0	0.64 (0.63-0.65)
SSC-PHF7-dif	0.45	0.46	0.48	0	0.46 (0.45-0.48)
SSC-TDRD1-end	0.63	0.64	0.65	0	0.64 (0.63-0.65)
SSC-MAN1A-end	0.55	0.56	0.57	0	0.56 (0.55-0.57)
SSC-SMC1B-end	0.55	0.56	0.57	0	0.56 (0.55-0.57)
SSC-LMO4-end	0.6	0.61	0.62	0	0.61 (0.6-0.62)
SSC-PHF7-end	0.43	0.45	0.46	0	0.45 (0.43-0.46)

Table S3. SSC- monkey_mouse_correlation

	95% CI Lower Limit	Correlation Coefficient	95% CI Upper Limit	P value	95% CI Summary
SSC-HMGN1-u1	0.53	0.54	0.56	0	0.54 (0.53-0.56)
SSC-STRA8-u1	0.46	0.48	0.49	0	0.48 (0.46-0.49)
SSC-CLGN-u1	0.24	0.25	0.27	1.56142145328725e-149	0.25 (0.24-0.27)
SSC-SDC4-u1	0.56	0.57	0.58	0	0.57 (0.56-0.58)

SSC-GSG1-u1	0.36	0.38	0.39	0	0.38 (0.36-0.39)
SSC-HMGN1-u2	0.46	0.47	0.49	0	0.47 (0.46-0.49)
SSC-STRA8-u2	0.41	0.42	0.44	0	0.42 (0.41-0.44)
SSC-CLGN-u2	0.22	0.24	0.26	4.65641114525116e-134	0.24 (0.22-0.26)
SSC-SDC4-u2	0.48	0.5	0.51	0	0.5 (0.48-0.51)
SSC-GSG1-u2	0.34	0.36	0.38	2.04932366175282e-310	0.36 (0.34-0.38)
SSC-HMGN1-u3	0.41	0.42	0.44	0	0.42 (0.41-0.44)
SSC-STRA8-u3	0.38	0.39	0.41	0	0.39 (0.38-0.41)
SSC-CLGN-u3	0.18	0.2	0.22	1.19645194289886e-90	0.2 (0.18-0.22)
SSC-SDC4-u3	0.43	0.44	0.46	0	0.44 (0.43-0.46)
SSC-GSG1-u3	0.27	0.29	0.31	4.82718608477216e-196	0.29 (0.27-0.31)
SSC-HMGN1-dif	0.63	0.64	0.65	0	0.64 (0.63-0.65)
SSC-STRA8-dif	0.57	0.58	0.59	0	0.58 (0.57-0.59)
SSC-CLGN-dif	0.33	0.35	0.36	6.89267699458207e-286	0.35 (0.33-0.36)
SSC-SDC4-dif	0.62	0.63	0.64	0	0.63 (0.62-0.64)
SSC-GSG1-dif	0.46	0.48	0.49	0	0.48 (0.46-0.49)
SSC-HMGN1-end	0.62	0.64	0.65	0	0.64 (0.62-0.65)
SSC-STRA8-end	0.58	0.59	0.61	0	0.59 (0.58-0.61)
SSC-CLGN-end	0.33	0.35	0.37	6.28615626760923e-294	0.35 (0.33-0.37)

SSC-SDC4-end	0.63	0.64	0.65	0	0.64 (0.63-0.65)
SSC-GSG1-end	0.46	0.48	0.49	0	0.48 (0.46-0.49)
